# Multifunctional Intelligent Hydrogels Based on MnO_2_ Nanozymes and Ca^2+^ Signal Regulation for Diabetic Wound Repair

**DOI:** 10.3390/gels12090826

**Published:** 2026-09-08

**Authors:** Yanling Li, Yuhan Mao, Ji’e Zhang, Lele Li, Rongfeng Zhao, Qian Pang, Fang Yang, Ruixia Hou

**Affiliations:** Department of Cell Biology and Regenerative Medicine, Health Science Center, Ningbo University, Ningbo 315211, China

**Keywords:** micelles, nanozymes, dynamic responsiveness, smart hydrogels, diabetic wound repair

## Abstract

Diabetic refractory wounds are a prevalent and severe complication of diabetes, whose pathological progression is jointly mediated by multiple factors, including oxidative stress imbalance, chronic inflammation, impaired angiogenesis, bacterial infection, and biofilm formation. Current clinical hydrogel dressings generally suffer from drawbacks such as single-function performance, potential toxicity of nano-components, static networks incompatible with dynamic wound conditions, and the absence of bionic repair signals. Therefore, they cannot simultaneously satisfy the dual repair requirements of complex pathological microenvironments and dynamic mechanical properties for diabetic wounds. In this study, a multi-functional dynamically responsive composite hydrogel (MC group) with high-efficiency antioxidant, antibacterial, and pro-angiogenic capacities was fabricated. Using SDS-C_18_ micelles as hydrophobic units, a rigid–flexible dual-network framework was constructed with polyvinyl alcohol (PVA) and methacrylated hyaluronic acid (HAMA). Manganese dioxide nanozymes were introduced to scavenge reactive oxygen species (ROS) and mitigate oxidative stress. Calcium-ion-mediated dynamic micelle reconstruction was adopted to regulate the hydrophilic–hydrophobic balance, while achieving antibacterial effects and facilitating tissue regeneration. In vitro experiments verified that the MC hydrogel possesses mechanical properties well-matched to human soft tissues (fracture stress: 25 kPa) and excellent biocompatibility (cell viability > 100%, hemolysis rate: only 0.13%). It also exhibits prominent antioxidant activity (DPPH radical-scavenging rate: 36.95%), antibacterial performance (>99.86% bactericidal rate against *Staphylococcus aureus*, survival rate of *Escherichia coli* reduced to 15.95%), and cell-migration-promoting activity (endothelial cell migration rate of 83.72% and mouse fibroblast migration rate of 90.88% within 24 h). In the full-thickness skin defect model of diabetic mice, the wound-healing rate reached 99% on day 16. Moreover, it promoted ordered collagen deposition, skin appendage regeneration, and functional microvascular reconstruction, thereby accomplishing high-quality tissue repair. This design synergistically intervenes in multiple pathological links of diabetic wounds, overcomes several key limitations of existing dressings, and provides an innovative strategy for developing smart dressings.

## 1. Introduction

Diabetes mellitus is a highly prevalent chronic metabolic disease worldwide with a continuously rising incidence. Diabetic refractory wounds represent one of the common and severe chronic complications of diabetes. Clinical data indicate that approximately 34% of patients with type I and type II diabetes develop cutaneous ulcers during disease progression [1]. Some non-healing wounds may further progress to deep-tissue infection and gangrene, ultimately leading to amputation, which substantially increases patient mortality risk and healthcare system burden [2].

The core mechanism underlying impaired diabetic wound healing lies in multi-factor-mediated blockade of the healing cascade under hyperglycemic pathological conditions. Specifically, excessive generation of local reactive oxygen species (ROS) induces oxidative-stress imbalance [3]; abnormal macrophage polarization sustains persistent chronic inflammation [4]; suppressed functions of endothelial cells and fibroblasts result in impaired angiogenesis [5] and insufficient collagen synthesis [6]; and recurrent bacterial infection [7] and the formation of drug-resistant biofilms further aggravate tissue injury. These interrelated pathological events constitute the hallmark features of refractory wounds, suggesting that an ideal therapeutic strategy should simultaneously target multiple pathological nodes. Conventional dressings remain the mainstay of current clinical management. Products such as gauze and medical foams merely provide physical coverage and exudate absorption and cannot actively reverse the chronic pathological microenvironment of wounds. Moreover, dressing changes tend to cause secondary injury to newly formed granulation tissue and exhibit poor conformability to irregular wound beds [8]. Accordingly, developing functional dressings that actively modulate multiple pathological pathways while adapting to dynamic mechanical variations in wounds represents an urgent unmet clinical need.

In recent years, the field of multifunctional wound-repair hydrogels has developed rapidly, giving rise to a variety of material design strategies. Layered double hydroxides (LDHs), benefiting from their tunable compositions and excellent drug-loading capacity, can promote fibroblast proliferation or regulate extracellular matrix remodeling by releasing metal ions such as Zn^2+^ and Mg^2+^ for skin wound repair [9]. Janus hydrogels achieve unidirectional drainage and functional zoning by virtue of asymmetric physicochemical properties on their two sides (adhesive on one side and anti-adhesive on the other) [10]. Motion-responsive lubricating hydrogels, with dynamic cross-linked networks, provide lubrication during tissue movement and form a barrier at rest to inhibit fibrotic pathways [11]. Nevertheless, the above-mentioned systems possess relatively single functions and can hardly synergistically address multiple pathological challenges, including oxidative stress, bacterial infection, and tissue repair, on a single material platform.

Targeting oxidative stress as a core pathological event, two major design strategies are currently adopted. The first category covers ROS-responsive hydrogels, into which oxidation-sensitive moieties, such as phenylboronate esters, thioethers, or thioketals, are commonly incorporated to achieve on-demand drug release or controllable network degradation under highly oxidative conditions [12]. For instance, the phenylboronate-based hydrogel reported by Qiao et al. [13] possesses favorable in situ responsiveness; nevertheless, its ROS-scavenging capacity is limited by the total amount of responsive groups. Moreover, mechanical performance decays upon network degradation, making long-term scavenging efficiency hard to sustain. The second category is nanozyme-based hydrogels, represented by MnO_2_ and CeO_2_. These hydrogels display catalase-like and superoxide dismutase-like activities, catalyzing ROS decomposition to produce O_2_ and thereby alleviating oxidative stress and local hypoxia [14]. A typical example is the CeO_2_ nanozyme-loaded hydrogel developed by Tian et al. [15]. However, nanoparticles tend to aggregate within hydrogel networks of high ionic strength, leading to deteriorated catalytic activity, and their single antioxidant function cannot satisfy infection control requirements.

In terms of antibacterial therapy and repair-signal modulation, Ca^2+^-mediated hydrogels have attracted growing attention. Ca^2+^ exerts broad-spectrum antibacterial effects by disrupting bacterial membranes and interfering with biofilm formation. Meanwhile, as a second messenger, it activates the migration, proliferation, and differentiation of keratinocytes and fibroblasts. Nevertheless, existing Ca^2+^-containing hydrogels mostly employ calcium alginate, calcium-crosslinked gelatin, or bioactive glass as carriers. A representative example is the Ca^2+^-crosslinked sodium alginate hydrogel fabricated by Liu et al. [16]. In such systems, Ca^2+^ merely serves as a cross-linker or a simple ion-releasing source. The dual biological functions of antibacterial activity and repair promotion have not been systematically integrated, accompanied by risks of burst ion release and excessive local calcium concentration. Given the above limitations, current studies fail to realize spatiotemporal synergy among catalytic antioxidation, ionic antibacterial/anti-biofilm effects, and pro-migration signals within a single hydrogel platform. In addition, the matching design between functional integration and dynamic mechanical networks is frequently overlooked.

**Scheme 1:**
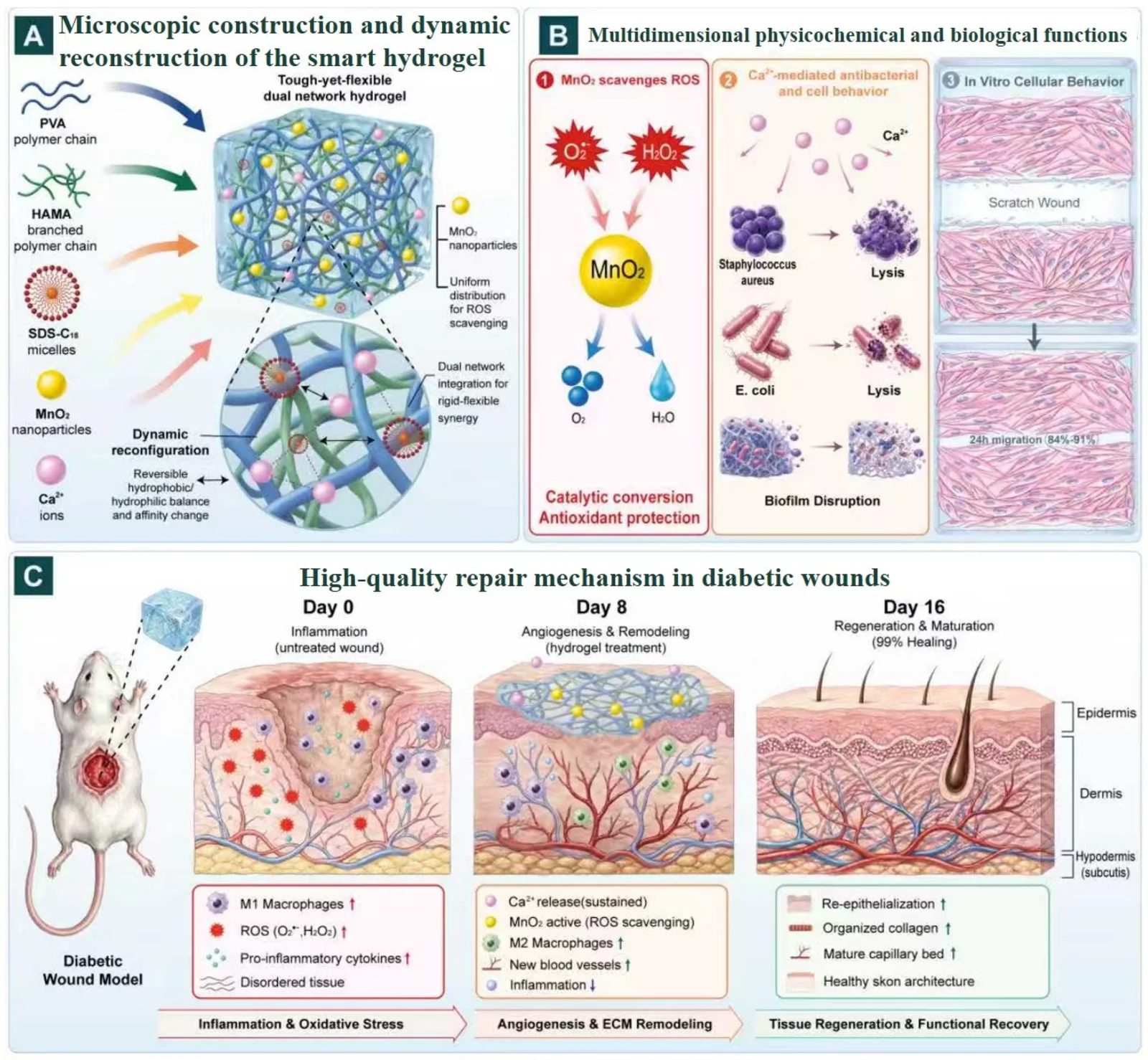
Schematic illustrations of the construction of MnO_2_/Ca^2+^ hydrogel and its mechanism for diabetic wound repair. (**A**) The tough-yet-flexible dual-network hydrogels were constructed from PVA, HAMA, SDS-C_18_ micelles, MnO_2_ nanoparticles, and Ca^2+^ ions. (**B**) The multifunctional hydrogel exerted antioxidant protection via MnO_2_ nanozymes, Ca^2+^-mediated antibacterial activity, and promoted cell migration in vitro. (**C**) The hydrogel accelerated diabetic wound healing in vivo by alleviating inflammation, promoting angiogenesis, and restoring healthy skin architecture.

To address these challenges, this work designs and constructs a multifunctional intelligent double-network hydrogel based on the MnO_2_ nanozyme and Ca^2+^ signal modulation for accelerating diabetic wound repair, as illustrated in Scheme 1. The hydrogel is assembled from poly(vinyl alcohol) (PVA), methacrylated hyaluronic acid (HAMA), SDS-C_18_ micelles, homogeneously dispersed MnO_2_ nanoparticles, and Ca^2+^. Among them, the dynamically reversible cross-linking system, built by interpenetrating double networks together with ionic coordination, hydrophobic interactions, and hydrogen bonds, endows the material with combined rigid–flexible mechanical properties and dynamic reconstructive capability. It can adapt to dynamic deformation of wound beds, firmly anchor MnO_2_ nanozymes to suppress aggregation, continuously catalyze ROS decomposition, and mitigate oxidative stress and hypoxia. Ca^2+^ modulates the hydrophilic–hydrophobic balance of SDS micelles and is integrated via dynamic coordination, performing both cross-linking and signal-regulating functions to achieve broad-spectrum antibacterial and anti-biofilm activities as well as promote cell migration. The above components collaboratively intervene in multiple pathological processes of diabetic wounds, accelerating wound healing and improving tissue repair quality. This provides new design insights for developing novel dressings for diabetic wounds.

## 2. Results and Discussion

**Figure 1:**
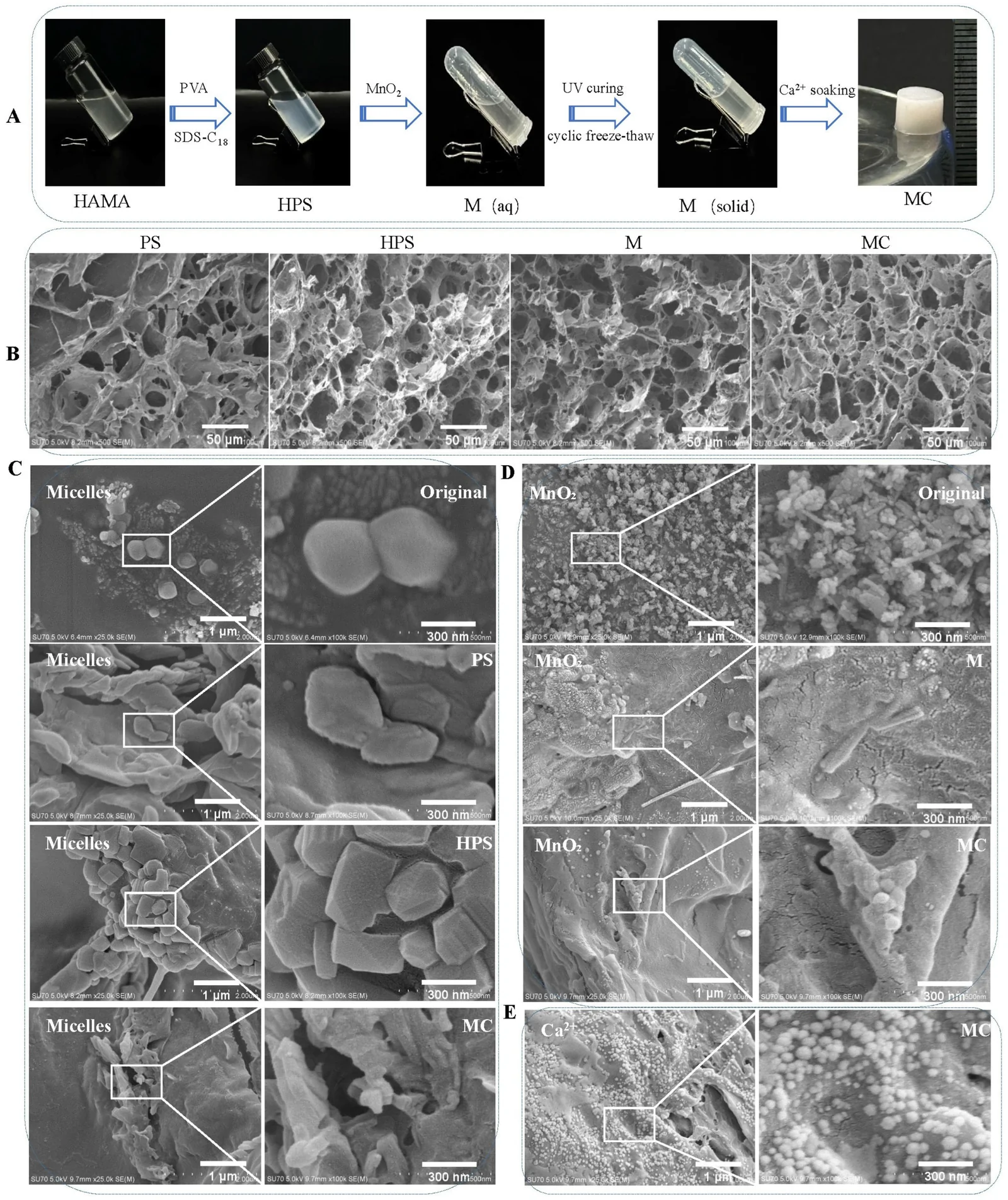
Preparation and morphological characterization of the multifunctional hydrogel. (**A**) Schematic illustration of the preparation process of the MC composite hydrogel. (**B**) Scanning electron microscopy (SEM) images of the internal three-dimensional porous network structure of the multifunctional composite hydrogels in different groups. (**C**) SEM images of SDS-C_18_ micelles in different groups. (**D**) SEM schematic diagram of MnO_2_ nanoparticle powder in different groups. (**E**) SEM image of the micromorphology of Ca^2+^ in the MC hydrogel (*n* = 5).

Aiming at the core clinical challenge of the complex microenvironment and persistently impaired healing process of diabetic skin ulcer wounds, this study designed an MC multifunctional composite hydrogel integrating multiple physicochemical properties and biological activities, whose preparation process and micromorphological characterization are shown in Figure 1A–E. The construction of the MC hydrogel started with the dual-network matrix. Using polyvinyl alcohol (PVA) and methacrylated hyaluronic acid (HAMA) as the interpenetrating dual-network skeleton, the core network was constructed through a stepwise cross-linking process of “UV curing first, followed by cyclic freeze-thawing”: UV irradiation triggered free radical polymerization of the methacrylate groups on the HAMA molecular chains [17], forming a covalently cross-linked rigid chemical network; cyclic freeze-thawing promoted hydrogen bond cross-linking between PVA molecular chains to form a flexible physical network. The two formed a “rigid–flexible integrated” physical–chemical dual-network structure through topological interpenetration, which endowed the hydrogel with excellent mechanical properties, ensuring that it could withstand the dynamic deformation of skin tissue and maintain the integrity of the three-dimensional structure during wound healing. Meanwhile, the hyaluronic acid matrix in HAMA, as a natural extracellular matrix component, synergistically improved the swelling stability, water retention, and biocompatibility of the hydrogel [18].

Sodium dodecyl sulfate-stearyl methacrylate (SDS-C_18_) micelles were used as the dynamic cross-linking functional units, which formed a core–shell micelle structure through the self-assembly mechanism of “hydrophilic head exposed and hydrophobic tail aggregated”. Meanwhile, they simultaneously anchored the carboxyl groups of HAMA and the hydroxyl groups of PVA via hydrogen bonding, significantly increasing the cross-linking density of the network. The surface of MnO_2_ nanozyme particles was rich in hydroxyl groups, which formed hydrogen bonds with the polar groups on the molecular chains of HAMA and PVA to avoid their agglomeration. The subsequent immersion treatment in Ca^2+^ solution further optimized the network: on the one hand, Ca^2+^ could specifically coordinate with the carboxyl groups on the uronic acid units of HAMA molecular chains, forming dynamic, reversible coordination cross-linking bonds, which further improved the cross-linking density and structural stability of the network. On the other hand, Ca^2+^ could bind to the sulfate groups on the surface of SDS-C_18_ micelles through electrostatic interaction, breaking the original hydrophilic–hydrophobic assembly balance of the micelles, promoting the partial exposure of the hydrophobic core of the micelles, and generating stronger hydrophobic interaction with the hydrophobic segments of PVA and HAMA in the network. This anchored the micelles from free physical cross-linking points to the reinforcement phase in the network, realized multi-scale synergistic cross-linking of chemical covalent cross-linking, hydrogen bonding, ionic coordination, and hydrophobic interaction, and significantly enhanced the structural stability and mechanical properties.

Microstructural characteristics are the structural basis for various properties of the hydrogel, and a uniform and dense pore structure is conducive to moisture management, substance transport, and mechanical stability [19]. Scanning electron microscopy (SEM) showed (Figure 1B) that the four groups of hydrogels, including PS (PVA/SDS-C_18_ single network), HPS (HAMA/PVA/SDS-C_18_ dual network), M (HAMA/PVA/SDS-C_18_/MnO_2_) and MC (HAMA/PVA/SDS-C_18_/MnO_2_/Ca^2+^), all presented a porous structure. However, with the improvement of the network structure and the introduction of functional components, there were significant inter-group differences in pore size distribution, pore connectivity, pore wall integrity, and network uniformity among each group. The PS group only relied on hydrogen bonds between PVA molecular chains to form physical cross-linking and lacked stable cross-linking point control during the gelation process, resulting in disordered and loose stacking of polymer chains, presenting a loose structure with large pore size, inhomogeneity, thin pore walls, and poor continuity. After the introduction of the HAMA chemical cross-linking network, the HPS group showed reduced pore size, more uniform distribution, thickened and continuous pore walls, forming a more stable three-dimensional porous network structure. The M group uniformly loaded MnO_2_ nanoparticles in the HPS dual-network system, which further optimized the network. There was no pore blockage or structural collapse caused by nanoparticle agglomeration, and the good porous structure of the dual-network hydrogel was still maintained. After Ca^2+^ treatment in the MC group, the coordination with the sulfate groups of SDS induced dynamic and ordered rearrangement of SDS-C_18_ micelles. Meanwhile, Ca^2+^ formed dynamic ionic cross-links with the carboxyl groups on the HAMA chains, introducing a large number of uniformly distributed dynamic reversible cross-linking sites on the basis of the dual network. Finally, a three-dimensional porous network structure with uniform pore size, highly connected pores, and continuous and complete pore walls was formed. Compared with the previous three groups, it had the best structural stability and uniformity, which was suitable for the rapid absorption of wound exudate, efficient transport of oxygen and nutrients, and infiltration and proliferation of repair cells, perfectly meeting the application requirements of dressings for diabetic wound repair.

The morphological evolution of micelles (Figure 1C) further revealed the network regulation effect. The original micelles (Original group) presented a uniform spherical-like morphology with uniform particle size (approximately 500 nm) and smooth surface. After being embedded in the PS single network, the hydrogen-bonded physical cross-linking network formed by freeze–thaw cycles underwent anisotropic shrinkage during freeze-drying, exerting non-uniform mechanical compressive stress on the micelles embedded in the network pores [20], resulting in the transformation of their morphology into an irregular flat block structure with a rough surface. In the HPS dual network, the micelles evolved into angular cubic-like blocks with ordered stacking. This ordered assembly structure was also well maintained in the M group hydrogel, which only introduced MnO_2_ nanoparticles without changing the main framework of the dual network, which was attributed to the uniform confinement effect constituted by the HAMA covalent network and the PVA physical network. After Ca^2+^ treatment in the MC group, the micelles underwent fundamental reconstruction: the original spherical micelle assemblies were completely dissociated, and the micelle units rearranged along a specific axis, presenting an elongated rod-like or fibrous structure. They intertwined with each other to form a loose three-dimensional network structure, which was enriched in the network cracks and pore channels of the hydrogel, forming a clear structural boundary with the surrounding polymer matrix. The mechanism lies in that the specific binding of Ca^2+^ to the sulfate groups (-OSO_3_^−^) on the micelle surface, on the one hand, neutralizes the negative charge on the micelle surface and significantly weakens the electrostatic repulsion between micelles; on the other hand, it compresses the hydration layer around -OSO_3_^−^ through the ionic hydration competition effect, reducing the hydrophilicity and steric hindrance of the hydrophilic end of the micelles [21], thus breaking the original hydrophilic–hydrophobic balance of the micelles, and making the hydrophobic interaction of the long alkyl chains of C_18_ the main attractive force between micelles. As a divalent cation, Ca^2+^ can form “ionic bridging” between adjacent micelles, enhance the binding strength, and guide the micelles to stretch, align, and fuse along a specific axis, and self-assemble to form an anisotropic fibrous structure. This fibrous network tends to be enriched in the network cracks and pore channels of the hydrogel because they provide a low-constraint free space for fiber extension and interweaving, which is more thermodynamically and structurally conducive to the formation of a stable porous topological structure.

The morphology and dispersion behavior of MnO_2_ nanoparticles (Figure 1D) showed that the original MnO_2_ powder presented a mixed morphology of spherical-like and short rod-like shapes with severe agglomeration. In the M group hydrogel, due to the spatial confinement effect of the three-dimensional porous network of the hydrogel [22] and the hydrogen bonding of the polymer chains, their agglomeration was effectively limited, but there was still an obvious interface boundary between the MnO_2_ particles and the hydrogel matrix, which was prone to the risk of particle shedding, burst release, and secondary agglomeration during the swelling and degradation of the hydrogel. After Ca^2+^ treatment in the MC group, MnO_2_ was uniformly anchored on the polymer segments of the hydrogel in a monodisperse or minimal aggregate form, forming a highly compatible integrated interface with the surrounding polymer matrix without obvious phase separation and interface boundary. This maximally exposed the catalytic active sites of MnO_2_ nanoparticles, ensured the efficient and sustained exertion of their superoxide dismutase (SOD)-like and catalase (CAT)-like activities, and avoided the cytotoxicity caused by the burst release of nanoparticles.

In the MC hydrogel, Ca^2+^ was tightly bound to the polymer matrix of the hydrogel in a regular, spherical-like, and monodisperse form, forming a compatible and continuous interfacial bond with the surrounding organic polymer network without obvious phase separation (Figure 1E). This highly uniform monodisperse distribution characteristic provides core structural support for global uniformity, synchronous sustained release of functional ions, and in situ synergy of multiple effects, and can effectively avoid asynchronous healing and excessive scar hyperplasia caused by uneven functional distribution of traditional dressings.

In this study, macroscopic visual characterization was performed to verify that the MC hydrogel possessed excellent injectability, in situ formability, and structural stability in the aqueous environment of wound exudate. Relying on its shear-thinning property, the hydrogel could be smoothly injected through a medical syringe for precise molding and rapidly achieved in situ cross-linking and curing in the aqueous environment mimicking wound exudate while maintaining long-term structural stability. The excellent performance of the hydrogel originated from the shear-reversible property [23] endowed by the full-network dynamic reversible cross-linking system constructed with the synergy of Ca^2+^, as well as the “rigid–flexible synergy” structure of the PVA/HAMA interpenetrating dual network, which solved the core drawback of traditional injectable hydrogels that are prone to swelling and disintegration in water-rich environments. Different from the motion-responsive hydrogel that only relies on disulfide bonds to achieve sol–gel transition for tendon lubrication, our multi-dynamic crosslinking system not only adapts to tissue movement, but also maintains structural stability and interface adhesion under a wet exudate environment, which is more suitable for irregular and dynamically changing diabetic wound beds. Meanwhile, compared with the prefabricated static Janus membrane, which is difficult to fit complex wound surfaces, the injectable in situ forming hydrogel has better clinical operability and conformability.

**Figure 2:**
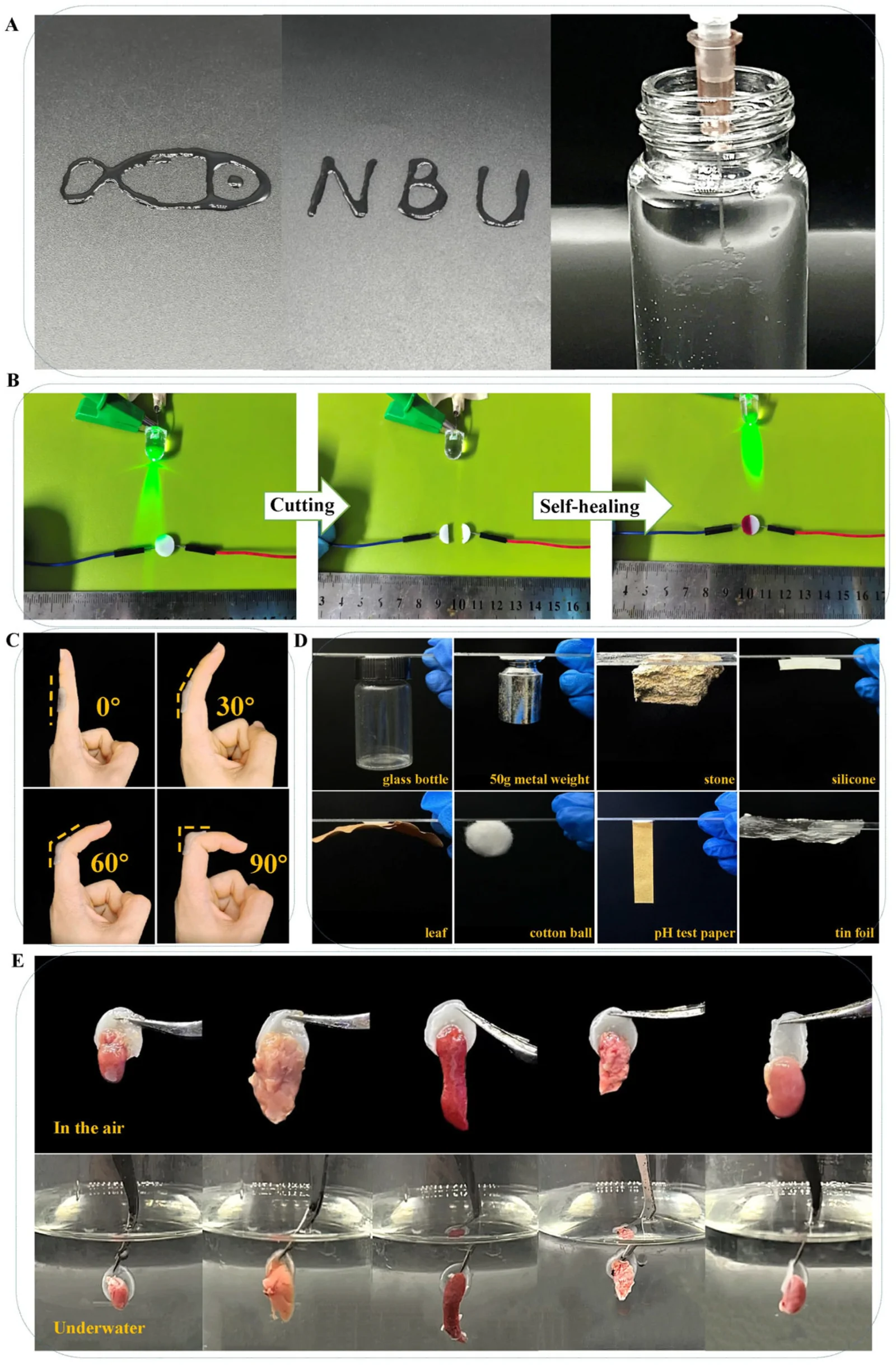
Schematic illustration of the injectability, electrical conductivity, self-healing property, and adhesiveness of the hydrogel. (**A**) Schematic illustration of the injectability of the MC hydrogel. (**B**) Electrical conductivity and self-healing process of the MC hydrogel. (**C**) Flexible adaptation performance of the MC hydrogel at different bending angles of the finger joint. (**D**) Interfacial adhesion effect of the MC hydrogel on various substrates in air. (**E**) Adhesion stability of the MC hydrogel to biological tissues in air and underwater (*n* = 5).

To verify the ionic conductivity, self-healing property, and recovery ability of conductive function after structural damage of the MC hydrogel, a closed circuit was constructed with the hydrogel as the conductive medium for evaluation (Figure 2B). When the intact MC hydrogel was connected to the circuit, the light-emitting diode (LED) was stably lit, indicating its favorable ionic conductivity. The conductive mechanism was attributed to the free directional migration of uniformly distributed mobile Ca^2+^ in the three-dimensional network through the porous network and hydration channels. After the hydrogel was completely cut along the direction perpendicular to the current with a surgical blade, the LED went out, and the conductive function was lost. The cut sections (one half was stained) were closely fitted, and after standing at room temperature for a period of time, the LED was re-lit with no difference in brightness from that before cutting, which demonstrated that the MC hydrogel could achieve synchronous and efficient self-recovery of both structural integrity and conductive function. Its self-healing property originated from the dynamic hydrophilic–hydrophobic association formed by Ca^2+^-induced micelle rearrangement and the non-covalent cross-linking system constructed by Ca^2+^-HAMA dynamic ionic cross-linking bonds. After the sections were fitted, the molecular chains realized interfacial fusion through free diffusion and entanglement, and the dynamic cross-linking bonds underwent reversible dissociation and reconstruction, enabling autonomous healing without external energy input and synchronously restoring the mechanical integrity and ion migration channels of the hydrogel. The combination of stable ionic conductivity and excellent self-healing properties provides support for the development of long-acting smart dressings and the realization of dynamic wound repair.

To meet the clinical requirements of tight fit and long-term sealing of diabetic wound dressings in complex application scenarios such as dynamic deformation and tissue exudation, the dynamic adhesion stability, broad-spectrum substrate adhesion ability, and tissue adhesion performance in different environments of the MC hydrogel were further evaluated (Figure 2C–E). The dynamic compliance test (Figure 2C) showed that during continuous bending from 0° to 90°, the hydrogel always maintained close contact with the skin tissue without edge lifting, slippage, or detachment, exhibiting excellent dynamic deformation adaptability and interfacial adhesion stability. The substrate adhesion performance test (Figure 2D) indicated that in the air environment, the MC hydrogel could firmly adhere to the surface of substrates with different polarity and roughness, including glass, metal, stone, silicone, leaves, cotton, pH test paper, and tinfoil, and could stably bear its own weight and the weight of the substrate without detachment, showing a universal interfacial bonding ability. Using fresh isolated biological soft tissues as the model (Figure 2E), the MC hydrogel could form tight and continuous contact with different tissues, including the heart, liver, spleen, lung, and kidney, both in air and in the underwater water-rich environment mimicking wound exudate, without interfacial separation or detachment.

The excellent adhesion performance of the MC hydrogel originated from a multi-dimensional synergistic interfacial mechanism. First, the molecular adhesion mechanism was based on the synergy of multiple types of non-covalent bonds. The hydroxyl groups on the PVA chains formed a global hydrogen bond network with the polar groups of the tissue; the carboxyl groups of HAMA and the sulfate groups of the micelles formed strong electrostatic interactions with the positively charged collagen peptide chains in the tissue; Ca^2+^ constructed ionic bridges between the carboxyl/phosphate groups of the hydrogel and the tissue, forming an integrated cross-linked structure between the hydrogel and the skin tissue to achieve high-strength interfacial adhesion. Second was the dynamic mechanical adaptation mechanism. The “rigid–flexible integrated” interpenetrating dual network and dynamic cross-linking structure endowed the material with excellent deformation ability, which buffered external dynamic stress through reversible deformation and maintained dynamic adhesion stability. Third was the interfacial water film removal and wound adaptation mechanism. The Ca^2+^-regulated dynamic hydrophilic–hydrophobic micelles removed interfacial water molecules through hydrophobic interaction, realizing direct contact between the hydrogel and the tissue surface [24]; the PVA/HAMA porous network rapidly absorbed excess exudate, promoting the molecular-level close contact between the polymer segments and the tissue surface. This multi-dimensional synergistic mechanism enables the MC hydrogel to achieve long-term adhesion under dynamic deformation, atmospheric conditions, and exudate-rich aqueous environments, broadening its application scenarios including skin wound repair, underwater biological sealing, and interfacial integration of flexible devices.

**Figure 3:**
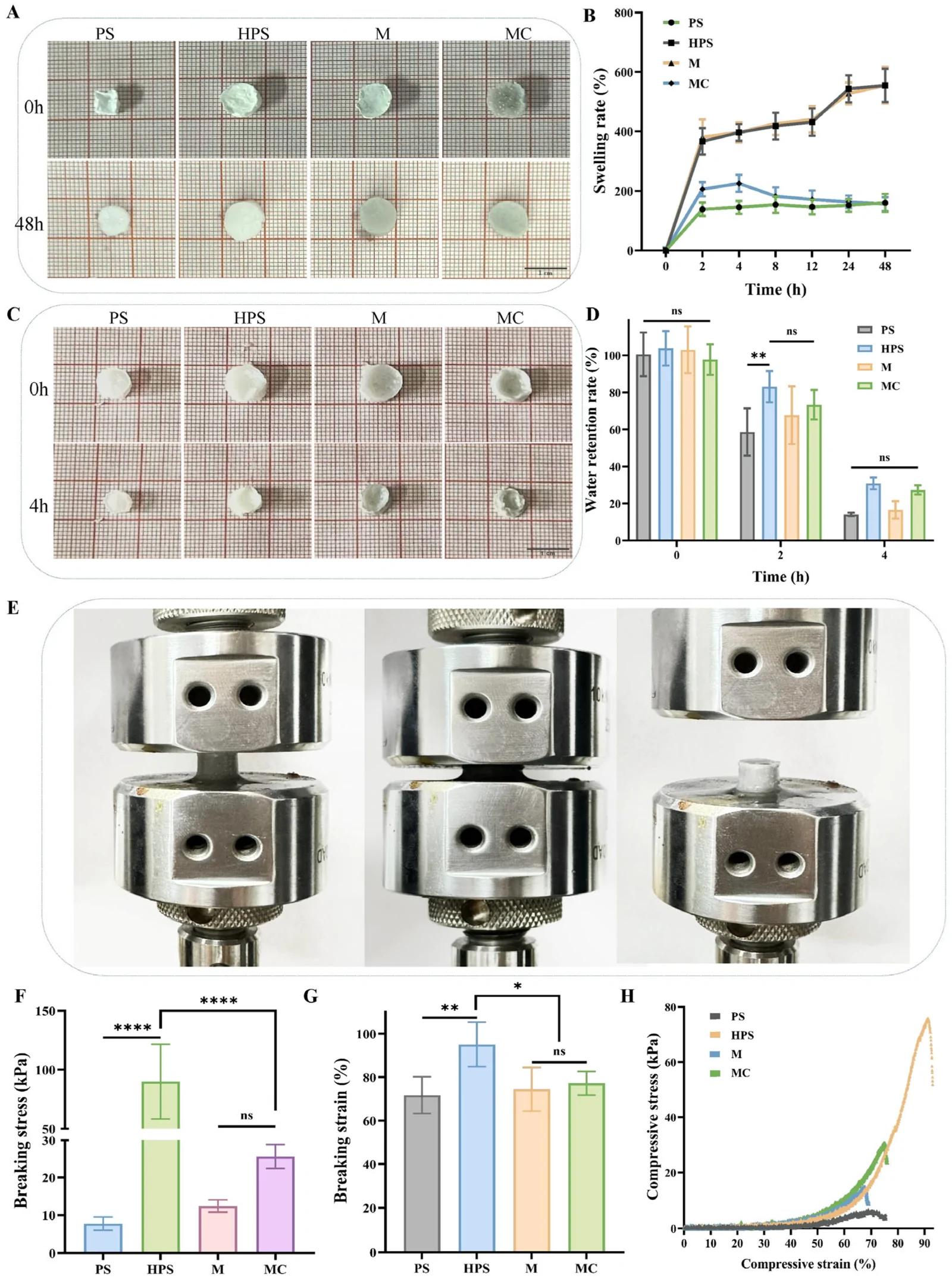
Evaluation of the physical properties of the multifunctional composite hydrogel. (**A**) Macroscopic swelling morphology of the multifunctional composite hydrogel. (**B**) Swelling kinetic curves of the multifunctional composite hydrogel. (**C**) Macroscopic water retention morphology of the multifunctional composite hydrogel. (**D**) Water retention rate results of the multifunctional composite hydrogel. (**E**) Physical image of the compression mechanical property test of the MC hydrogel. (**F**) Tensile strength at break of different multifunctional hydrogels. (**G**) Tensile strain at break of different multifunctional hydrogels. (**H**) Stress–strain curves of the multifunctional hydrogels (*n* = 5). * *p* < 0.05, ** *p* < 0.01, **** *p* < 0.0001, ns means no significant.

Swelling property is a core fundamental performance of wound dressings, which directly determines its wound exudate absorption capacity, three-dimensional structural stability, interfacial fit, and the ability to maintain a moist healing microenvironment. An ideal diabetic wound dressing should have moderately controllable swelling capacity, which can not only rapidly absorb excess exudate and reduce the risk of infection, but also avoid structural collapse and fitting failure caused by excessive swelling, while maintaining a stable swelling equilibrium throughout the entire wound healing cycle [25]. For this purpose, in this study, phosphate-buffered saline (PBS) at pH 7.4 was used to mimic the physiological wound microenvironment, and the macroscopic structural stability and swelling kinetics of the four groups of hydrogels (PS, HPS, M, and MC) were systematically evaluated (Figure 3A,B).

At the initial stage of swelling, the PS single-network hydrogel presented an irregular block structure with curled edges and poor surface flatness; the HPS, M, and MC groups all showed a disk-shaped morphology with intact structure, regular edges, and a uniform and dense surface, indicating that the interpenetrating dual network significantly optimized the structural uniformity of the hydrogel after freeze-drying. After 48 h, the PS group showed an extremely low degree of volume expansion and severely insufficient adsorption capacity; the HPS and M groups showed significant excessive swelling, with a greatly increased disk diameter, fluffy and softened texture, and insufficient anti-swelling constraint capacity; while the MC group only underwent moderate volume expansion with intact and uniform structure, no loosening, curling or collapse, exhibiting excellent structural stability during the swelling process. The quantitative analysis was consistent with the macroscopic morphology. The equilibrium swelling rate of the PS group at 48 h was only 160% because the dense and stable hydrophobic association cross-linked network formed strong spatial constraints on the expansion of molecular chains, which significantly limited the infiltration of water molecules. The swelling kinetics of the HPS and M groups were highly consistent, showing a trend of rapid and continuous swelling without equilibrium constraint, with the 48 h equilibrium swelling rates reaching 562% and 559%, respectively. This was attributed to the high hydrophilicity and water storage space endowed by a large number of hydrophilic carboxyl groups on the HAMA molecular chains and the interpenetrating porous structure, while the lack of an effective swelling constraint mechanism led to excessive swelling. The MC group presented an ideal swelling kinetic behavior of rapid water absorption and swelling in the early stage, autonomous retraction equilibrium in the middle stage, and long-term stable maintenance in the later stage. Specifically, the swelling rate rapidly reached 206% within 2 h, peaked at 225% at 4 h, then gradually decreased, and stabilized at 156% at 24 h, showing excellent swelling equilibrium regulation ability and long-term structural stability. Its anti-swelling performance was significantly better than that of traditional static hydrogel dressings (e.g., the dual-crosslinked N-isopropylacrylamide-based hydrogel dressing designed by Cao et al. [26], which could reach 2250% at 12 h), and was comparable to high-performance anti-swelling materials (e.g., the high-performance anti-swelling film (RBVATHF) specially designed for underwater electronic skin by Lin et al. [27], with a swelling rate of 165%).

The ideal swelling characteristics of the MC hydrogel originated from the synergistic regulation of the multi-level dynamic cross-linked network. The hydrophilic skeleton and three-dimensional porous structure of the PVA/HAMA interpenetrating network ensured the rapid response and absorption capacity to wound exudate; the dynamic ionic cross-linking formed by Ca^2+^ and the carboxyl groups of HAMA, as well as the dynamic hydrophilic–hydrophobic cross-linking formed by Ca^2+^-induced micelle rearrangement, precisely regulated the overall cross-linking density and established a dynamic balance between swelling and constraint; the dynamic reversible cross-linking bonds maintained the network integrity through dissociation and reconstruction during the swelling process. For clinical application, the rapid initial swelling capacity can absorb excess exudate in a timely manner, reducing the risk of exudate maceration and infection. The long-term stable equilibrium swelling rate can maintain a moderately moist microenvironment during the slow healing process, avoiding dry scab formation and secondary mechanical damage and frequent dressing changes caused by dressing replacement.

Water retention property determines the ability of the dressing to maintain a moist healing microenvironment, which is closely related to the wound healing process. In this study, hydrogels at swelling equilibrium were used to evaluate the water retention performance of the four groups of samples in an open environment at room temperature (Figure 3C,D). At 0 h, all four groups of hydrogels presented a full, hydrated disk shape with regular edges. After standing for 2 h, the water retention rate of the PS group decreased most significantly (*p* < 0.05), while there was no significant difference among the HPS, M and MC groups, indicating that the interpenetrating dual network improved the short-term water holding capacity of the hydrogel, and the MnO_2_ nanoparticles and Ca^2+^ dynamic cross-linking system had no negative impact on the short-term water retention performance of the hydrogel. After standing for 4 h, there was no statistically significant difference in the water retention rate among the groups (*p* > 0.05), but the macroscopic morphology was significantly different. The PS group underwent severe irreversible shrinkage, with a shriveled and hardened structure, completely losing the structural and functional integrity of the hydrogel; the HPS and M groups only showed mild and uniform shrinkage and maintained a regular disk shape. This morphological stability originated from the physical adsorption of hydrophilic groups on the HAMA molecular chains and the water-locking effect of the interpenetrating network [28], but such static rigid networks cannot achieve adaptive adjustment with changes in the external environment and wound state. In contrast, the MC group showed wavy shrinkage at the edges and overall convergence. The mechanism lies in the Ca^2+^-regulated dynamic SDS-C_18_ micelle structure has environmental response characteristics. When water content decreases, the hydrophobic interaction around the micelles is enhanced, driving microphase separation and macroscopic gradient shrinkage, thus forming an irregular morphology. For clinical application, the MC group has both moderate water retention capacity and adaptive shrinkage characteristics, which not only avoid dry scab formation and exudate accumulation, but also achieve synchronous deformation with the healing shrinkage of the wound, maintain full interface fit, and form a synergy with the adhesion performance.

Mechanical properties are the core foundation for the clinical application of dressings. The excellent compression mechanical properties can ensure that the diabetic wound dressing maintains the integrity of the three-dimensional network structure, provides continuous and stable physical barrier protection for the wound, and avoids mechanical compression and secondary damage to the fragile new granulation tissue of the wound, especially adapting to the complex mechanical application scenarios of wounds in joint moving parts. The uniaxial compression test (Figure 3E) showed that the MC hydrogel deformed uniformly under large strain compression without brittle fracture, and recovered to its initial shape after the external force was removed, possessing good elastic deformation capacity and structural reversibility.

Further quantitative analysis showed that the fracture stress of HPS was increased to 89.9 kPa, and the fracture strain reached 94.8%, both of which were significantly higher than those of the other three groups (*p* < 0.0001). This was attributed to the continuous stress transfer path constructed by the PVA/HAMA interpenetrating dual network through topological entanglement and multi-dimensional cross-linking, which effectively dissipated the mechanical stress during compression. The fracture strains of the PS, M, and MC groups were all in the range of 71~77%, with no significant difference among the groups, but there was a significant gradient difference in the compressive fracture stress among the groups. The PS group had the lowest compressive stress of only 7.7 kPa, reflecting that the rigid physical cross-linked network relying only on hydrogen bonds lacked an effective stress dissipation mechanism; the compressive fracture stress of the M group was (12.36 ± 2.34) kPa, and its mechanical properties were significantly deteriorated compared with the HPS group. The reason was that the electrostatic binding between the charged sites on the surface of MnO_2_ nanoparticles and the carboxyl groups of HAMA molecules occupied a large number of cross-linking active sites, leading to a decrease in the effective cross-linking density of the interpenetrating dual network and the destruction of the stress transfer path. The compressive stress of the MC group was 25.6 kPa, which was within the typical bearing range of physiological soft tissues (10–200 kPa) [29], closely matching the mechanical properties of human skin tissue, and significantly better than similar wound repair hydrogel materials (e.g., the methacrylate-modified chitosan-ferulic acid-oxidized *Bletilla striata* polysaccharide (CSOB-FA) hydrogel developed by Zhong et al. [30], with a fracture stress of 12 kPa). The mechanism lies in the Ca^2+^-mediated dynamic cross-linking system introduced uniformly distributed reversible sacrificial bonds to construct an efficient stress dissipation structure.

The mechanical response characteristics in the full strain range (Figure 3H) showed that in the low-medium strain range of 0~70% (corresponding to the typical strain range of daily skin activities such as limb flexion and extension), the compressive stress of each group was maintained at a low level from nearly 0 to about 10 kPa; in the high strain range of 70~90% (corresponding to the scenario of large deformation of the skin or external force action), each group showed obvious differentiation. The stress of the HPS group rose sharply, reaching nearly 80 kPa at 90% strain; the PS and M groups were always lower than 10 kPa. The former was difficult to resist structural collapse under large deformation due to the lack of sufficient entanglement and a rigid skeleton, while the latter had significantly insufficient mechanical bearing capacity because MnO_2_ destroyed the original interpenetrating network structure and load transfer path. In contrast, the MC group presented a gradient response characteristic adapted to the needs of diabetic skin ulcers: it maintained low stress compliance in the 0~70% strain range to avoid the impact of daily mechanical stimulation; in the high strain range of 70~90%, the compressive stress increased moderately, reaching about 30 kPa at 90% strain. This response originated from the synergistic effect of reversible dissociation of dynamic cross-linking bonds to dissipate stress and appropriate support provided by topological entanglement of the interpenetrating dual network, enabling the material to maintain structural integrity under extreme deformation while avoiding damage to fragile tissues due to excessive stress.

**Figure 4:**
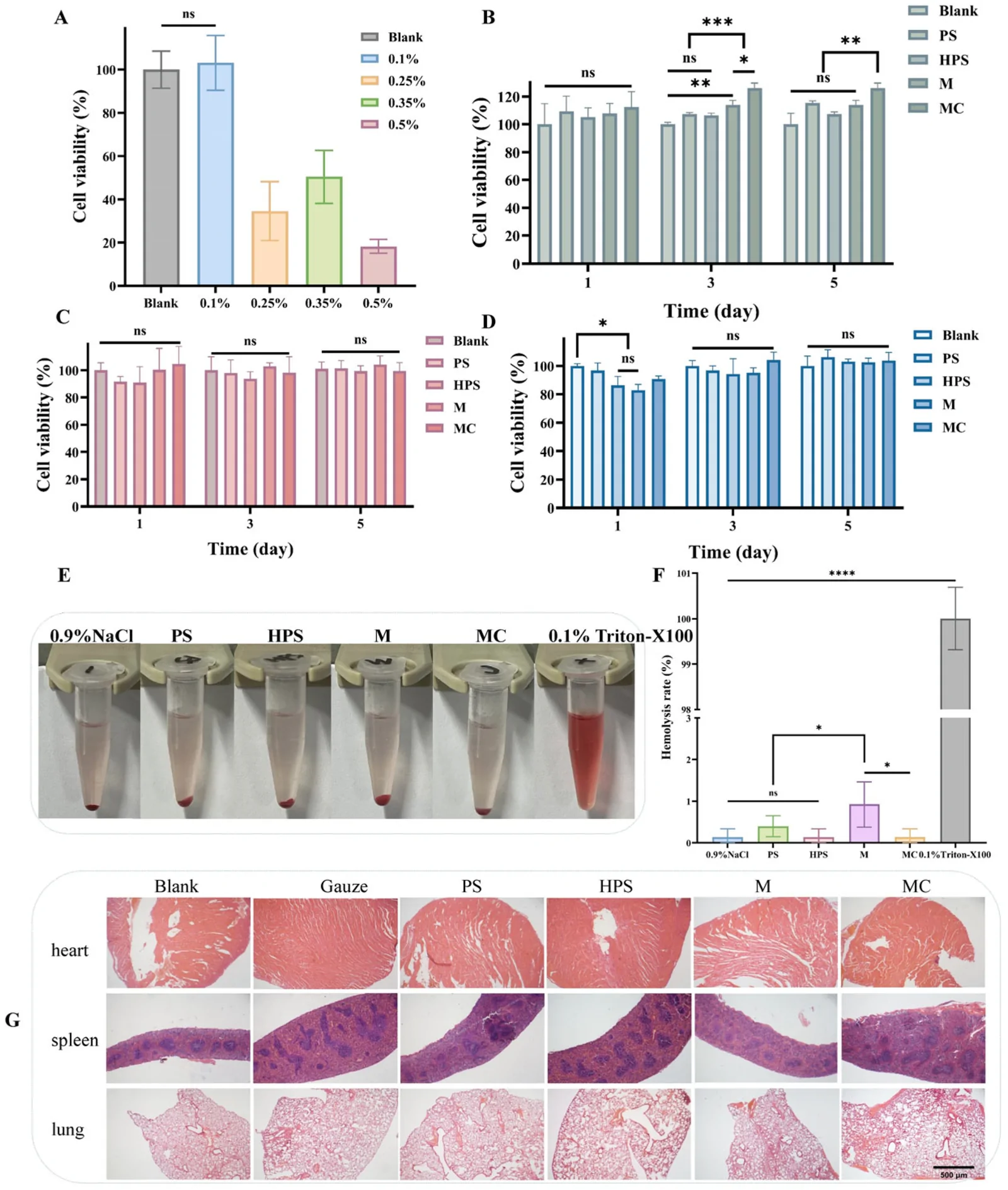
Biocompatibility evaluation of the multifunctional composite hydrogel. (**A**) Cell viability of human umbilical vein endothelial cells (HUVECs) treated with composite hydrogels at different MnO_2_ concentrations (0.1%, 0.25%, 0.35%, 0.5%). (**B**) Cell viability of endothelial cells co-cultured with the multifunctional composite hydrogel on days 1, 3, and 5. (**C**) Cell viability of fibroblasts co-cultured with the multifunctional composite hydrogel on days 1, 3, and 5. (**D**) Cell viability of macrophages co-cultured with the multifunctional composite hydrogel on days 1, 3, and 5. (**E**) Macroscopic images of the in vitro hemolysis test of the multifunctional composite hydrogel. (**F**) Hemolysis rate of hydrogels in each group. (**G**) Hematoxylin and Eosin (H&E) histopathological staining of major organs (heart, spleen, lung) after in vivo implantation of the materials (*n* = 5). * *p* < 0.05, ** *p* < 0.01, *** *p* < 0.001, **** *p* < 0.0001, ns means no significant.

Cytocompatibility is the core prerequisite for the clinical translation and application of medical biomaterials. In this study, the CCK-8 assay was used to systematically evaluate the safe application concentration of the MC multifunctional composite hydrogel, as well as its compatibility and biological activity regulatory effect on wound repair-related functional cells and immune cells via the indirect contact method with extracts. The relevant results are shown in Figure 4. The results in Figure 4A showed that the cell viability of the 0.1% concentration group was 103%, with no significant difference from the blank control group (*p* > 0.05, ns), indicating that the MC hydrogel had no obvious cytotoxicity at this concentration. With the increase in extract concentration, the cell viability showed a concentration-dependent decrease (the cell viability of the 0.25%, 0.35%, and 0.5% concentration groups dropped to 35%, 50%, and 18%, respectively), all of which were significantly lower than that of the blank control group. The reason is that high concentrations of MnO_2_ nanoparticles easily enter cells through endocytosis [31] and generate excessive reactive oxygen species (ROS) intracellularly; disrupt the intracellular redox homeostasis; and cause mitochondrial damage, oxidative DNA damage, and destroy cell membrane integrity. Therefore, the safe concentration of 0.1% was used in all subsequent in vitro and in vivo experiments to avoid potential cytotoxic risks.

Figure 4B shows the cell viability of human umbilical vein endothelial cells (HUVECs) after co-culture with 0.1% safe-concentration extracts of four groups of hydrogels (PS, HPS, M, and MC) for 1, 3, and 5 days, which was used to evaluate the long-term cytotoxicity and pro-proliferative activity of the materials toward endothelial cells. After 1 day of co-culture, the cell viability of each group was approximately 100%, with no significant difference compared with the blank control group (*p* > 0.05). After 3 days of co-culture, the cell viability of each group increased to varying degrees. Among them, the MC group achieved a cell viability of 110%, which was significantly higher than the control group, PS group, and HPS group (*p* < 0.001). The cell viability of the M group was also significantly higher than that of the control group, indicating that a low concentration of the MnO_2_ nanozyme can effectively scavenge excess reactive oxygen species and alleviate oxidative-stress-induced damage. After 5 days of co-culture, the cell viability of each group was further elevated, and the MC group still maintained a cell viability above 120%, which was significantly higher than that of the blank control group. The above results demonstrate that the MC hydrogel realizes the slow and controllable release of bioactive components MnO_2_ and Ca^2+^ via its dynamic cross-linked network. Meanwhile, the degradation products of hydrogel matrix materials PVA and HAMA are non-cytotoxic, providing a favorable microenvironment for the proliferation and physiological function of endothelial cells.

Fibroblasts are the core functional cells for granulation tissue formation, collagen deposition, and tissue remodeling in skin wound repair, and often show impaired proliferative activity and insufficient extracellular matrix synthesis during the pathological progression of diabetic skin ulcers [32]. Figure 4C shows the cell viability of the four groups of hydrogels co-cultured with NIH/3T3 fibroblasts for 1, 3, and 5 days. During the entire co-culture period, the cell viability of each group was stably maintained above 90%, with no significant difference from the blank control group (*p* > 0.05), indicating that the PVA and HAMA matrix materials have good biocompatibility without the release of toxic components, meeting the biosafety requirements of medical wound dressings. The cell viability of the MC group showed a steady upward trend with the culture time, which was attributed to the fact that low-concentration sustained release of Ca^2+^ from the dynamic cross-linked network can regulate collagen synthesis and proliferation of fibroblasts.

Macrophages are the primary responders to the host immune response after material implantation, and their viability state directly affects the intensity of the inflammatory response and the efficiency of the tissue integration of the material [33]. Figure 4D shows the cell viability of RAW 264.7 murine monocyte-macrophages co-cultured with the extracts of the four groups of hydrogels for 1, 3, and 5 days. On day 1 of culture, the macrophage viability of the HPS group (86%) and the M group (83%) showed a temporary decrease, which was significantly lower than that of the control group (*p* < 0.05), while the MC group (91%) showed good initial compatibility. On the basis of the HPS group, the M group introduced the MnO_2_ nanozyme. Inorganic nanoparticles with high surface energy are easily recognized as foreign bodies by macrophages, further triggering a phagocytic stress response, resulting in a more significant decrease in viability. The MC group retained the active domain of hyaluronic acid through dynamic ionic cross-linking between Ca^2+^ and the carboxyl groups of HAMA, which can exert the immune homeostasis regulatory effect mediated by the CD44 receptor. Meanwhile, the dynamically cross-linked network endows the material with low-modulus flexible characteristics that better match physiological soft tissues, which greatly reduces the initial foreign body stress and mechanical stress of macrophages. On days 3 and 5 of co-culture, the macrophage viability of each group recovered to above 95%, with no significant difference from the blank control group (*p* > 0.05), confirming that none of the four hydrogel groups had continuously released toxic components or had good long-term immune compatibility. In summary, the MC multifunctional composite hydrogel at the safe concentration of 0.1% fully meets the biosafety requirements of medical biomaterials without cytotoxicity. Compared with the PS, HPS, and M groups, the MC hydrogel has both excellent cytocompatibility and pathological targeted functional adaptability. It can effectively promote the proliferation of vascular endothelial cells, support the normal physiological activity of fibroblasts, and alleviate the initial foreign body stress of macrophages.

The hemolysis test is a core indicator to evaluate the safety of the interaction between biomaterials and blood [34]. In this study, a red blood cell suspension was prepared from fresh whole blood of ICR mice, and the hemolytic performance of the four groups of hydrogels was systematically evaluated by the indirect contact method with extracts (0.9% NaCl solution was used as the negative control, and 1% Triton X-100 solution was used as the positive control). The relevant results are shown in Figure 4E. The supernatant of the positive control group was uniformly dark red, while the supernatants of the negative control and the experimental groups (PS, HPS, M, MC) were all clear and transparent, with no visible hemoglobin release. The quantitative hemolysis rate (Figure 4F) showed that the hemolysis rate of the negative control was 0.13%, and that of the PS group, HPS group, and MC group was 0.39%, 0.13%, and 0.13%, respectively, all of which were far below the 5% safety threshold specified in the national standard [35] and were not statistically different from the negative control group. The hemolysis rate of the PS group was slightly increased because the PVA single network is only cross-linked by hydrogen bonds with a relatively loose structure, and there are a small number of hydrophobic microdomains on the surface. The hemolysis rate of the M group was 0.93%, which was slightly higher than that of other experimental groups but still far below 5%. The mechanism is that a very small amount of free nanoparticles is released from the static cross-linked network, which has a weak electrostatic interaction with the red blood cell membrane and very slightly upregulates the membrane permeability [36]. The hemolysis rate of the MC group was completely consistent with that of the negative control group. This is because it uses hydrophilic medical polymers PVA and HAMA as the matrix material, forming a dense hydration interface on the surface without destroying the integrity of the red blood cell membrane. In addition, the introduction of Ca^2+^ not only strongly anchors MnO_2_ nanoparticles and eliminates the slight hemolysis risk caused by free particles, but also fixes hydrophobic segments and avoids the increase in hemolysis rate caused by hydrophobic microdomains, thus having higher blood safety.

H&E staining for histocompatibility is the core pathological indicator to evaluate the in vivo application safety of biomaterials. In this study, based on the full-thickness skin defect model of diabetic ICR mice, the mice were treated with sterile gauze (gauze group), PS, HPS, M, and MC hydrogel dressings, respectively, and ICR mice without modeling and intervention were used as the blank control group (control group). After the intervention cycle, the heart, spleen, and lung tissues were collected for H&E staining (Figure 4G). The morphology of the heart, spleen, and lung tissues in the control group, gauze group, PS, HPS, M, and MC groups all maintained a normal physiological state, with no significant pathological differences among the groups. The myocardial fibers were arranged regularly with clear striations, no inflammatory infiltration, necrosis, or interstitial fibrosis; the structure of splenic lymphoid follicles was clear, the boundary between red and white pulp was distinct, with no abnormal hyperemia, hyperplasia, or atrophy; and the alveolar structure was intact with normal septal thickness, no inflammatory exudation or collapse. The results showed that the four groups of hydrogel dressings did not cause systemic inflammatory response or pathological damage to major organs during in vivo application, and had excellent in vivo histocompatibility.

Its in vivo safety is derived from the following aspects: polyvinyl alcohol (PVA) is an FDA-approved medical dressing material [37] with no toxic functional groups, which can be completely excreted with metabolism in vivo; methacrylic anhydride-modified hyaluronic acid (HAMA) is derived from the natural extracellular matrix, and its degradation product is hyaluronic acid oligosaccharides, which can be completely metabolized by hyaluronidase; MnO_2_ nanoparticles are anchored to the network through electrostatic interaction, and are only slowly released locally to avoid systemic exposure; Ca^2+^ is an essential major element of the human body, and after local release, it is rapidly regulated by the calcium homeostasis system. In addition, this study verified in the diabetes pathological model that none of the hydrogel dressings caused pathological damage to organs, confirming that they also have good histocompatibility and clinical translation value in pathological environments such as diabetic wounds.

**Figure 5:**
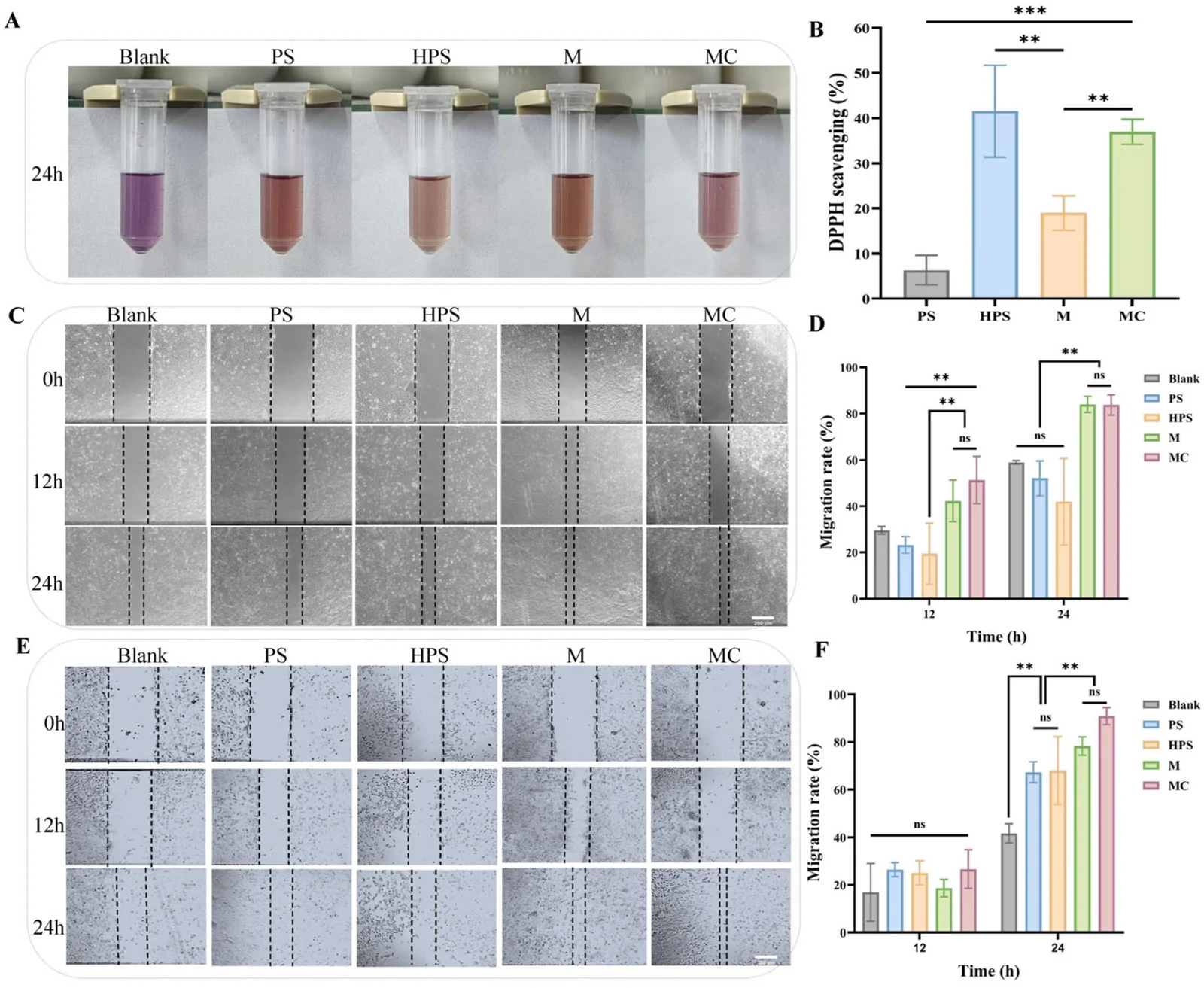
Evaluation of the antioxidant activity and in vitro cell migration ability of the multifunctional composite hydrogel. (**A**) Color development images of the DPPH free radical scavenging assay of the composite hydrogel. (**B**) Quantitative analysis of the DPPH free radical scavenging rate of the composite hydrogels in each group. (**C**) Images of the scratch wound migration assay of human umbilical vein endothelial cells (HUVECs) at 0 h, 12 h, and 24 h. (**D**) Quantitative statistics of the scratch wound migration rate of endothelial cells treated with the composite hydrogel. (**E**) Images of the scratch wound migration assay of fibroblasts. (**F**) Quantitative statistics of the scratch wound migration rate of fibroblasts (*n* = 5). ** *p* < 0.01, *** *p* < 0.001, ns means no significant.

The 517 nm absorbance and the purple color fade after being reduced, which can quantitatively reflect the antioxidant capacity of the material. As shown in Figure 5A,B, after co-incubation with the four groups of hydrogels, the DPPH solution showed different degrees of purple fading, among which the HPS group and the MC group had the most significant change. The PS group had the lowest scavenging rate of only 6% because it was only cross-linked by hydrogen bonds of polyvinyl alcohol (PVA) and lacked high-density active sites. The scavenging rate of the HPS group was significantly increased to 42%, which was significantly higher than that of the PS and M groups (*p* < 0.001) and was attributed to the large number of retained hydroxyl and carboxyl groups on the HAMA molecular chains that could act as hydrogen donors to reduce free radicals. The scavenging rate of the M group was only 19% because the electrostatic adsorption of MnO_2_ nanoparticles shielded the active sites of HAMA. The MC group achieved a scavenging rate of 37%, which was significantly higher than that of the PS group and M group (*p* < 0.001), with no significant difference from the HPS group, and a 9-fold significant increase compared with the antioxidant efficiency of pure HAMA hydrogel (4%) reported in previous studies [38]. The mechanism is that the dynamic coordination between Ca^2+^ and the carboxyl groups of HAMA competitively reduces the shielding of active sites by MnO_2_, retains the hydrogen donor function of HAMA, and realizes the dual-mechanism synergy of broad-spectrum free radical scavenging by HAMA and specific enzymatic antioxidant activity of MnO_2_.

Angiogenesis is the core link to reverse wound ischemia and hypoxia and promotes the transition from the inflammatory phase to the proliferative phase, and the directional migration of endothelial cells is the primary prerequisite for the formation of new blood vessels [39]. In this study, the scratch wound healing assay was performed using HUVECs as the model to evaluate the regulatory effect of the four groups of hydrogels on endothelial cell migration (Figure 5C,D). After 12 h of intervention, the migration rates of the control group, PS group and HPS group were all lower than 30%, with no significant difference among the groups (*p* > 0.05); the scratch width of the M group and MC group was significantly narrowed, and the migration rate of the MC group reached 51%, which was significantly better than that of the PS group and HPS group (*p* < 0.01). After 24 h of intervention, the scratch in the MC group was almost completely closed, with a migration rate of 84%, which was significantly higher than that of the groups without MnO_2_ (*p* < 0.001), and the performance was superior to that of the hyaluronic acid hydrogel encapsulating mesenchymal stem cell-derived exosomes (HA-A/HA-SH@Exo) designed by Yu et al. [40]. The putative mechanism is that the MnO_2_ nanozyme scavenges reactive oxygen species to relieve the inhibition of the cytoskeleton induced by oxidative stress. Meanwhile, sustainably released Ca^2+^ from the MC group acts as a second messenger to activate signaling pathways, including PI3K/Akt and FAK, synergistically enhancing the short-term migratory effect.

Oxidative stress and inflammatory response inhibit the migration ability of fibroblasts and hinder the formation of granulation tissue. The fibroblast scratch wound assay (Figure 5E,F) showed that after 12 h of intervention, the migration rates of all groups were lower than 30%, with no significant difference among the groups (*p* > 0.05). The difference in short-term response compared with endothelial cells was attributed to the fact that fibroblast migration is more dependent on long-term biological processes such as extracellular matrix synthesis and focal adhesion maturation [41]. After 24 h of intervention, there was a significant gradient of differentiation among the groups. The blank group had the lowest migration rate of 41.6%; the PS group (67.3%) and HPS group (68%) constituted the basic pro-migration level, which was significantly higher than the blank group (*p* < 0.01); and the M group (78.3%) and MC group (90.9%) constituted the high-efficiency level, which was significantly better than the blank group, PS group, and HPS group (*p* < 0.01). Among them, the MC group showed the optimal pro-migration efficiency, and its 24 h cell migration rate was significantly better than the highest pro-migration rate (84.88%) of chitosan-polyvinyl alcohol based dual-network hydrogel (CSAEC/P50) on NIH/3T3 cells reported by Guo et al. [42].

**Figure 6:**
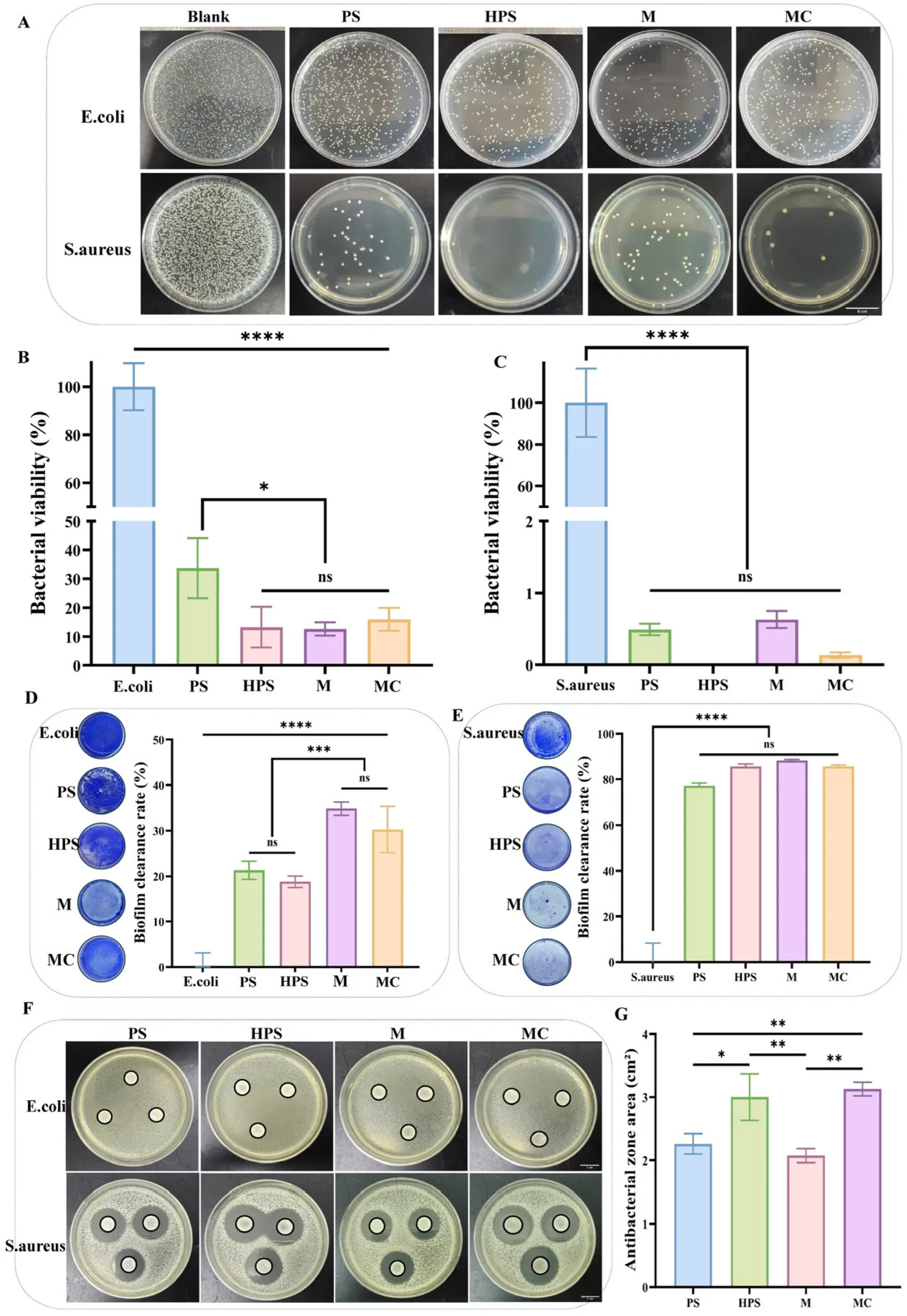
In vitro antibacterial performance of the multifunctional composite hydrogel. (**A**) Morphology images of viable colonies of *Escherichia coli* (*E. coli*) and *Staphylococcus aureus* (*S. aureus*) after 24 h of co-incubation. (**B**) Quantitative analysis of the viable bacterial rate of *E. coli* after 24 h. (**C**) Quantitative analysis of the viable bacterial rate of *S. aureus* after 24 h. (**D**) Biofilm clearance effect and quantitative analysis of *E. coli* biofilms. (**E**) Biofilm clearance effect and quantitative analysis of *S. aureus* biofilms. (**F**) Morphology of the inhibition zones against *E. coli* and *S. aureus*. (**G**) Quantitative analysis of the inhibition zone area against *S. aureus* (*n* = 5). * *p* < 0.05, ** *p* < 0.01, *** *p* < 0.001, **** *p* < 0.0001, ns means no significant.

Wound infection, especially the formation of drug-resistant biofilms, is a key factor leading to persistent inflammatory response, impaired healing, and even systemic infection. *Escherichia coli* (*E. coli*) and *Staphylococcus aureus* (*S. aureus*), the most common Gram-negative and Gram-positive bacteria in wound infections, are prone to form mature biofilms with high drug resistance, which can evade host immunity and antibiotic killing. Therefore, the development of dressings with broad-spectrum antibacterial activity, mature biofilm clearance capacity, and long-term bacteriostatic activity is essential for blocking infection and promoting wound healing. In this study, the antibacterial performance of the four groups of hydrogels was systematically evaluated. The bactericidal effect on planktonic bacteria, mature biofilm clearance capacity, and contact-sustained release bacteriostatic activity of the materials against *E. coli* and *S. aureus* were evaluated by plate coating colony counting assay, crystal violet staining biofilm clearance assay, and agar diffusion inhibition zone assay, respectively (Figure 6).

The plate coating colony counting assay (Figure 6A–C) showed that for *E. coli*, the PS group had the weakest antibacterial effect with a bacterial survival rate of 33.7%; the survival rates of the HPS, M and MC groups were approximately 13–16%, with no significant difference among the three groups (*p* > 0.05), and all were significantly lower than those of the control group and PS group (*p* < 0.0001). For *S. aureus*, the survival rate of all hydrogel groups was <1%, showing a nearly complete inhibitory effect. The antibacterial effect of the four hydrogel groups on *S. aureus* was better than that on *E. coli*. The mechanism is that the negatively charged carboxyl groups on the HAMA molecular chains can damage the bacterial cell membrane through electrostatic interaction, while the outer membrane structure of the Gram-negative bacterium *E. coli* can resist the invasion of external antibacterial components to a certain extent [43]. The MnO_2_ nanozyme catalyzes the generation of reactive oxygen species (ROS) through its peroxidase-like activity, which exerts a synergistic bactericidal effect with the electrostatic interaction of HAMA. The sustained-released Ca^2+^ in the MC group can destroy the integrity of the outer membrane lipopolysaccharide (LPS) of *E. coli*, enhance the inhibitory effect on *E. coli*, and ensure the broad-spectrum antibacterial performance of the material.

The crystal violet-stained biofilm eradication assay (Figure 6D,E) demonstrated that for mature *Escherichia coli* biofilms, the eradication rates of the PS and HPS groups were only approximately 20%, while those of the M and MC groups were 34.9% and 31.8%, respectively. No significant difference was observed between the M and MC groups (*p* > 0.05, ns), yet both values were significantly higher than those of the control, PS, and HPS groups (*p* < 0.001). For Staphylococcus aureus biofilms, the eradication rates of the PS, HPS, M, and MC groups all exceeded 77%, with no significant inter-group differences (*p* > 0.05, ns). These results suggest that the improved eradication efficiency against *E. coli* biofilms is mainly attributed to the introduction of the MnO_2_ nanozyme, whereas the addition of Ca^2+^ yielded no statistically detectable additional benefit under the present experimental conditions. A plausible explanation is that total biomass removal in the in vitro static biofilm model is dominated by MnO_2_-mediated oxidative killing, and the modulatory effect of Ca^2+^ on biofilms is relatively mild and fails to produce statistically significant differences at the tested exposure duration and concentration. Furthermore, crystal violet staining only reflects changes in total biofilm biomass [44]. If Ca^2+^ primarily acts on biofilm structural stability [45], intracellular bacterial viability, or biofilm re-formation capacity rather than total biomass, no obvious distinction between the two groups would be observed using this assay. For *S. aureus* biofilms, the electrostatic effect originating from the PVA/HAMA matrix already exerts considerable destructive potency, leaving limited room for further improvement by functional components.

The agar diffusion inhibition zone assay (Figure 6F,G) indicated that for *E. coli*, none of the four groups of hydrogels formed a visible inhibition zone. The reason is that HAMA has a large molecular weight and weak diffusion ability, and the active efflux system of *E. coli* can excrete low-concentration antibacterial substances. For *S. aureus*, all hydrogel groups formed clear inhibition zones. Among them, the inhibition zone area of the MC group was 3.1 cm^2^, which was significantly larger than that of the PS group and M group (*p* < 0.01), and was 1.55 times that of the kelp silver nanoparticle spray hydrogel (LamPBA/PVA-AgNPs) designed by Abbasi et al. [46] (inhibition zone area of approximately 2 cm^2^). Mechanistically, the PS group only relies on a physical barrier, with a limited antibacterial range. The M group depends on local ROS effect, which is difficult to form an inhibition zone through long-distance diffusion; the carboxyl groups of HAMA in the HPS group are fully exposed, which can migrate in the agar medium to form an antibacterial area; and the rapidly diffusing Ca^2+^ in the MC group can preferentially migrate to the distal end to pre-weaken the stability of the bacterial cell membrane, synergistically enhancing the antibacterial effect of the subsequently released HAMA. Meanwhile, the dynamic reversible binding between Ca^2+^ and HAMA avoids the permanent shielding of active sites, endowing the material with optimal long-term sustained-release bacteriostatic activity.

In summary, through the multi-mechanism synergy including the inherent electrostatic bacteriostasis of HAMA, ROS-mediated bactericidal effect of MnO_2_ nanozyme, and bacterial quorum sensing inhibition by Ca^2+^, the MC multifunctional composite hydrogel achieves efficient killing of planktonic *E. coli* and *S. aureus*, potent clearance of mature biofilms, and long-term sustained-release bacteriostasis, which perfectly adapts to the pathological characteristics of diabetic refractory wounds prone to infection and drug-resistant biofilm formation.

On the day of modeling (day 0), there was no significant difference in the initial wound area of each group, which was approximately 1.6 cm^2^. On day 4 (inflammatory phase), the blank group maintained a complete defect state with a healing rate of only 27.2%; the PS and HPS groups only showed slight contraction with no obvious granulation tissue regeneration; the gauze group formed dry scabs through physical isolation, and the M group scavenged ROS via MnO_2_ nanozyme, with their healing rates increased to 49.1% and 52.6%, respectively. The MC group achieved a healing rate of 66.6%, which was significantly higher than that of the blank, PS, HPS, and gauze groups (*p* < 0.001), indicating that it could effectively reverse the healing arrest of diabetic wounds at the early stage of repair. On day 8 (proliferative phase), the inter-group differences were further expanded. The blank group still had a large area of defect with stagnant re-epithelialization; the PS group had residual necrotic tissue with slow repair; and the wound area of the gauze group and HPS group was reduced, with healing rates of 78.4% and 71.4%, respectively, and no significant difference between the two groups. The wound of the MC group contracted significantly. The scabs basically fell off without exudation and residual necrotic tissue, and the healing rate was as high as 84.8%, significantly leading all other groups. In the remodeling phase, the growth rate of healing slowed down in all groups, but there were significant differences in repair quality. On day 16, the healing rates of the blank, gauze, PS, HPS, and M groups all exceeded 90%, but they left problems such as depressed scars, hyperpigmentation, rough epidermis, or sparse hair, respectively, and the repair stayed at the stage of incomplete regeneration. In contrast, the MC group achieved nearly perfect repair: the wound had naturally fused with the surrounding skin on day 12 (healing rate of 92.5%), and the healing rate reached as high as 98.7% on day 16, which was significantly better than the C70 fullerene/sodium alginate/acrylamide (AHF@AS) hydrogel designed by Sun et al. [47] (reaching 77.51% on day 14). The wound of the MC group was identical to normal skin in terms of color, texture, hair coverage, and regeneration of skin appendages, realizing high-quality regeneration in a true sense.

The essence of the above healing differences lies in the different regulatory abilities of each group of dressings on the pathological microenvironment. The blank, PS, and HPS groups only provide basic moisturizing or a physical barrier, lacking the ability to actively regulate oxidative stress, chronic inflammation, and infection risk; although the gauze group can isolate external contamination, it is easy to form dry scabs that hinder epithelial crawling and has no active pro-repair function. The MnO_2_ nanozyme loaded in the M group and MC group can scavenge excess ROS, inhibit the inflammatory cascade, and exert broad-spectrum antibacterial effects, reversing the healing arrest from the root cause. Among them, the MC group synergistically enhanced the effects of promoting cell migration, collagen deposition, and angiogenesis through the Ca^2+^-mediated dynamic cross-linked network, realizing multi-dimensional synergistic regulation of the pathological microenvironment of diabetic wounds, and ultimately driving the rapid and high-quality healing of diabetic refractory wounds.

**Figure 7:**
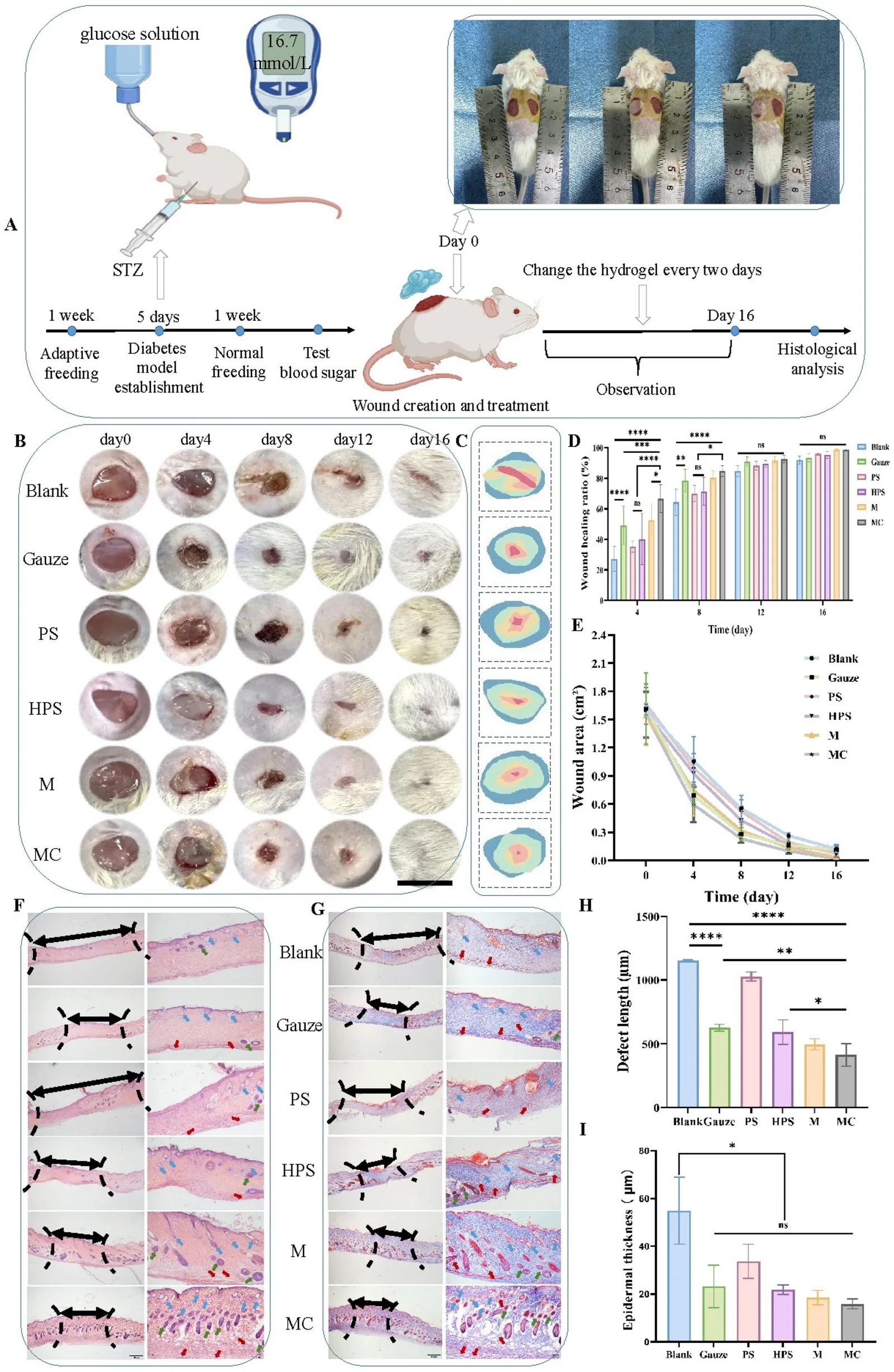
In vivo repair effect evaluation of the composite hydrogel on skin defect wounds in diabetic mice. (**A**) Schematic illustration of the experimental procedure for the diabetic mouse model establishment and wound treatment. (**B**) Wound healing images of mice in different groups on days 0, 4, 8, 12, and 16. (**C**) Heatmap of wound closure degree of each group corresponding to Figure (**B**). (**D**) Quantitative statistics of wound healing rate of different experimental groups from day 0 to 16. (**E**) Quantitative analysis of wound area of each group from day 0 to 16. (**F**) Hematoxylin–Eosin (H&E) histopathological staining sections of wound tissues of each group on day 16. (**G**) Masson’s trichrome histopathological staining sections of wound tissues of each group on day 16. (**H**) Quantitative statistics of defect length on day 16. (**I**) Quantitative statistics of epidermal thickness on day 16 (*n* = 5). * *p* < 0.05, ** *p* < 0.01, *** *p* < 0.001, **** *p* < 0.0001, ns means no significant.

Histopathological staining is the gold standard for evaluating the quality of wound repair. In this study, full-thickness skin tissues of the wounds in each group were collected on day 16 after modeling, and Hematoxylin–Eosin (H&E) staining and Masson’s trichrome staining were performed, respectively. The H&E staining results (Figure 7F) showed that the blank group had the longest epithelial defect length of approximately 1154.3 μm, which was 1.84 to 2.79 times that of the other effective intervention groups. The epidermal thickness was significantly increased to 54.9 μm, far exceeding the level of normal skin (approximately 20.57 μm) [48]. A large number of inflammatory cells infiltrated the dermis, the collagen fibers were arranged disorderly, and there were no mature capillaries or hair follicle structures. The repair state of the PS group was not significantly improved compared with the blank group; only the epidermal thickness was reduced (approximately 0.61 times that of the blank group). The epithelial defect length of the gauze group was reduced to 628 μm, the epidermal thickness was reduced to 23.1 μm, and the inflammatory cell infiltration was significantly reduced, but the collagen fibers were still loosely and disorderly distributed without mature skin appendages. The re-epithelialization process of the HPS group was similar to that of the gauze group; the defect length and epidermal thickness were recovered to the normal physiological range, and the regularity of collagen fiber arrangement was improved. Only sporadic hair follicle-like structures were observed. In the M group, the inflammatory infiltration basically subsided, the collagen fibers were slender and orderly arranged, a rich and mature capillary network was formed, and multiple early regenerated hair follicle germs were visible, indicating that the MnO_2_ nanozyme can relieve the inhibition of hair follicle stem cells caused by oxidative stress and activate the regeneration of skin appendages by scavenging ROS and inhibiting chronic inflammation. The wound of the MC group showed the optimal tissue repair effect, with complete re-epithelialization and the shortest defect length of only 413.20 μm, which was significantly shorter than that of all other groups (*p* < 0.0001). The epidermal layer was clear and complete, with a thickness reduced to 15.9 μm, consistent with normal skin; there was no obvious inflammatory cell infiltration in the dermis, and a large number of braided and orderly arranged collagen fibers, as well as abundant new capillaries and hair follicle-like skin appendages, were observed. These results indicate that the MC multifunctional composite hydrogel can promote complete re-epithelialization and orderly remodeling of the dermis through the synergistic effect of the MnO_2_ nanozyme and Ca^2+^, realizing high-quality tissue regeneration.

Masson’s trichrome staining was performed to further evaluate collagen deposition and maturity. The results showed (Figure 7G) that the dermis of the blank and PS groups had very few blue collagen fibers, which were sparse, slender and extremely disorderly arranged; the collagen fiber content of the gauze and HPS groups was increased, but still dominated by slender nascent collagen with loose and disordered arrangement; the M group had a significantly increased collagen fiber content, with thickened and regularly arranged fibers and a small number of collagen bundles visible; and the dermis of the MC group showed a large number of dense, thick, braided and orderly arranged blue collagen fibers, with the highest collagen deposition amount and maturity among all groups. These results further confirm that the MC hydrogel can significantly promote collagen maturation and orderly deposition in the dermal layer of the wound.

In summary, the MC multifunctional composite hydrogel prepared in this study can significantly promote complete re-epithelialization of diabetic wounds, orderly collagen deposition in the dermis, and regeneration of skin appendages, effectively reverse the tissue remodeling disorder of diabetic wounds, and achieve high-quality regenerative repair of the wounds, providing core histopathological evidence for its clinical translation and application.

**Figure 8:**
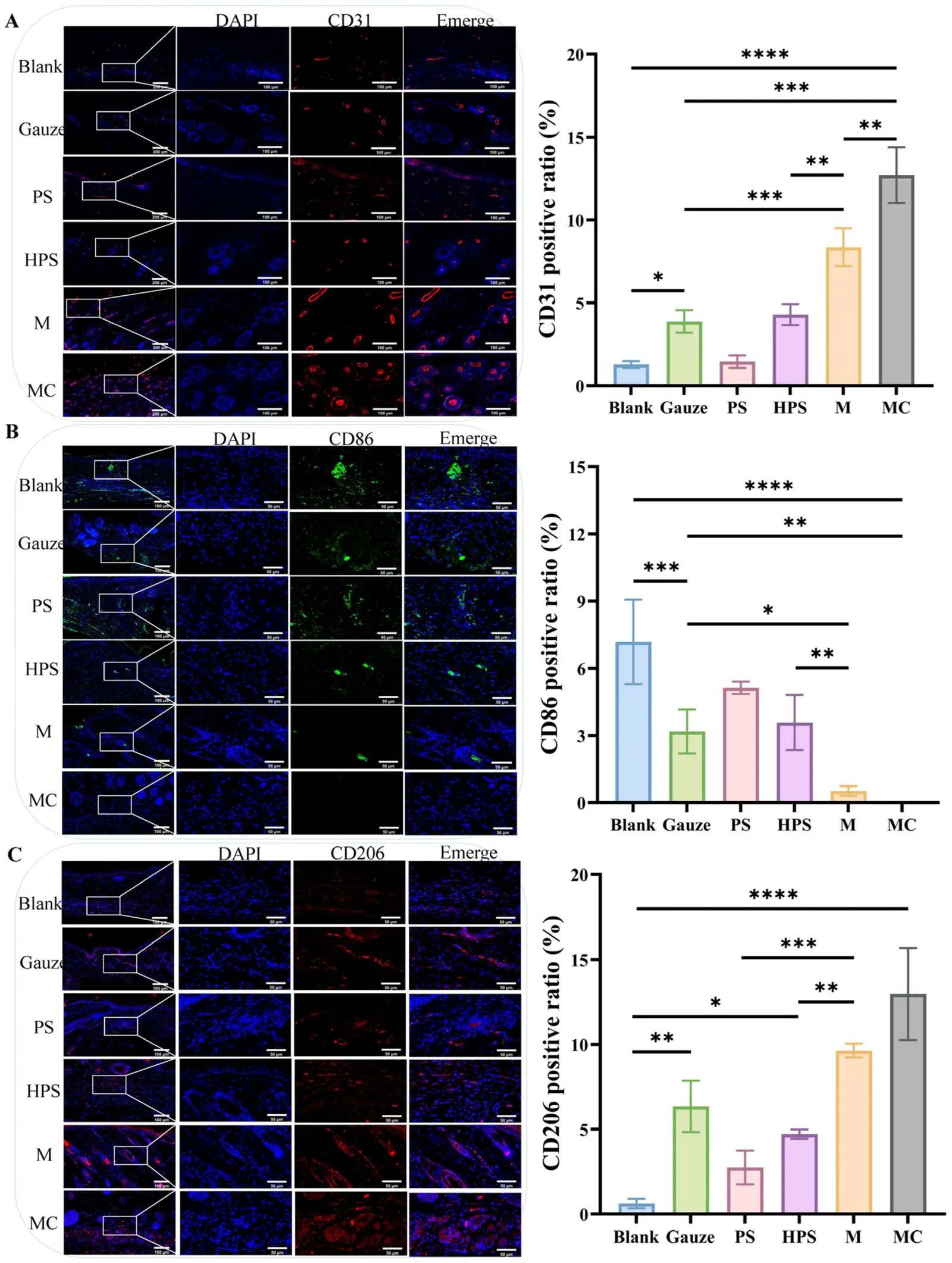
Immunofluorescence staining results of wound tissues in each group on day 16. (**A**) Representative images of CD31 immunofluorescence staining of wound tissues. (**B**) Representative images of CD86 immunofluorescence staining of wound tissues (CD31 in red, DAPI in blue). (**C**) Representative images of CD206 immunofluorescence staining of wound tissues (CD86 in green, CD206 in red, DAPI in blue) (*n* = 3). * *p* < 0.05, ** *p* < 0.01, *** *p* < 0.001, **** *p* < 0.0001.

To explore the mechanism by which this composite hydrogel modulates diabetic wound healing, systematic analyses were performed from the perspectives of angiogenesis and immunomodulation using immunofluorescence staining, and the relevant results are shown in Figure 8. Full-thickness skin tissues of wounds from each group were harvested on day 16 after model establishment. CD31 was used to label neovascular endothelial cells (red) and DAPI to label cell nuclei (blue), so as to observe the number of newly formed blood vessels, luminal structures, and vascular network formation. Only sporadic fragments of CD31-positive endothelial cells without intact luminal structures were observed in the blank group, indicating that the hyperglycemic microenvironment markedly inhibited angiogenesis. The CD31 signal was slightly enhanced in the gauze group, where a small number of tiny closed lumens could be seen; nevertheless, blood vessels were scarce and discretely distributed. The PS group exhibited sparse punctate CD31 signals with lower signal density than the gauze group. The HPS group showed significantly higher CD31 signal density than the PS and gauze groups, with more circular lumens and preliminarily formed vascular networks. This may be associated with the specific binding between HAMA and the CD44 receptor [49], which could promote endothelial cell adhesion, proliferation, and migration. Meanwhile, the favorable water-retention and moisturizing properties of the PVA/HAMA interpenetrating dual network may help maintain a moist wound microenvironment and mitigate inflammatory inhibition. In the M group, CD31 presented dense strip-shaped signals, and tortuously extending intact lumens were visible. The continuity and complexity of the vascular network were obviously improved, covering most areas of the wound. However, DAPI-labeled nuclei around blood vessels were scattered, suggesting insufficient infiltration of perivascular supporting cells and limited vascular stability. The MC group displayed the most favorable pro-angiogenic tendency. Quantitative analysis of CD31 immunofluorescence further confirmed this observation, with higher fluorescence intensity and vascular density relative to the PS, gauze, and HPS groups. Under low-magnification observation, CD31 signals reached the highest density with even distribution, and numerous interconnected circular and branched intact lumens constructed a dense microvascular network close to the physiological state of normal skin. Under high-magnification observation, closed circular mature lumens and C-shaped transitional structures were identified, and DAPI-labeled nuclei were densely distributed around blood vessels, implying the probable formation of functional blood vessels with certain stability. These results suggest that the pro-angiogenic effect of MC hydrogel may stem from its synergistic mechanism: the MnO_2_ nanozyme may scavenge ROS and alleviate inflammation, while Ca^2+^ may participate in the regulation of signaling pathways such as PI3K/Akt/eNOS and influence VEGF expression. However, whether these pathways are directly activated still requires further experimental validation.

In addition, immunofluorescence staining was conducted with CD86 (green) and CD206 (red), and DAPI (blue) was applied for nuclear labeling to observe the polarization status and distribution of macrophages. Macrophage polarization phenotypes exhibited distinct gradient differences among groups, and the expression of CD86 and CD206 showed a significant inverse correlation (Figure 8B,C). Wounds in the blank group presented a typical polarization-imbalanced phenotype: CD86 signals were densely clustered with high fluorescence intensity, whereas CD206 signals were extremely weak, suggesting blocked M2-type polarization. No obvious improvement was found in the PS group, where CD86 signals remained widely distributed. The gauze and HPS groups exerted comparable regulatory effects; CD86-positive signals were markedly attenuated, yet CD206 signals were only slightly elevated with limited improvement, which may be attributable to the merely physical barrier provided by gauze and the absence of loaded antioxidant components in HPS. In the M group, CD86 signals almost vanished, while abundant CD206 signals were densely distributed in continuous cord-like and mass-like patterns. This observation indicates that the MnO_2_ nanozyme may scavenge ROS, thereby potentially suppressing NF-κB-mediated M1 pro-inflammatory polarization and upregulating M2-polarization-related pathways, including STAT6/PPAR-γ [50]. Nevertheless, direct evidence supporting this mechanism is still lacking. Among all groups, the MC group achieved the optimal polarization-regulatory effect, and quantitative immunofluorescence results of CD86 and CD206 further corroborated this observation. CD86 green fluorescence nearly disappeared, and CD206 red fluorescence possessed the highest intensity and densest distribution, appearing as large-area continuous mass-like and reticular patterns. Positive cells spread throughout the whole wound-repair region and exhibited high co-localization with DAPI signals. Such superiority may benefit from the enhanced antioxidant regulation of MnO_2_ by the dynamic reversible cross-linked network. Meanwhile, Ca^2+^ may serve as a second messenger to participate in the crosstalk between the calcineurin-NFAT pathway and the STAT6 pathway [51], synergistically facilitating the expression of M2-phenotype-related genes. However, these pathway-related mechanisms have not been directly verified in the present study and need further confirmation via pathway-inhibition assays, protein-expression detection, and other approaches. Collectively, these results indicate that the MC multifunctional composite hydrogel possesses the potential to modulate the immune microenvironment of diabetic wounds and accelerate the healing of refractory wounds.

## 3. Conclusions

In view of the multiple pathological barriers in the clinical repair of diabetic skin ulcers and the key limitations of existing hydrogel dressings, this study successfully constructed an MC multi-functional dynamically responsive composite hydrogel integrating antioxidant, antibacterial, and pro-angiogenic functions. The hydrogel possesses a three-dimensional porous network with uniform pore size and favorable pore connectivity. It exhibits mechanical properties well-matched to human physiological soft tissues (compressive stress of 25 kPa), as well as intelligent swelling behavior characterized by rapid water absorption-autonomous retraction balance, injectability, self-healing capacity, and broad-spectrum adhesiveness. In vitro experiments demonstrate its favorable biocompatibility (cell viability > 100%, hemolysis rate of 0.13%), antioxidant activity (37% DPPH radical-scavenging rate), capability to promote the migration of endothelial cells and fibroblasts (24 h migration rates of 84% and 91%, respectively), and activity to inhibit *Staphylococcus aureus* and *Escherichia coli,* together with biofilm eradication. In the full-thickness skin-defect model of diabetic mice, via multi-mechanism synergy between the MnO_2_ nanozyme and Ca^2+^ signaling, the MC hydrogel actively modulates wound oxidative stress, inflammatory response, and angiogenesis, achieves a wound-healing rate of 99% on day 16, and facilitates ordered collagen deposition, skin appendage regeneration, and reconstruction of functional microvascular networks, showing the potential to shift from scar-mediated healing toward functional regeneration.

Nevertheless, the present study still has several limitations, including insufficient data on long-term biosafety, in vivo biodegradation, systemic toxicity and immune responses; unresolved challenges in storage stability, large-scale manufacturing and clinical translation; inadequate exploration of underlying molecular mechanisms and release kinetics; and potential bias in the reported wound closure rate caused by the contraction-dominated healing pattern of mouse cutaneous wounds. Future research will focus on systematic long-term toxicological and immunological assessments, elucidation of in vivo degradation pathways and release profiles, in-depth investigation of molecular regulatory mechanisms, optimization of storage conditions and scalable production processes, and validation in large-animal models that better mimic human wound healing, to further verify the efficacy and safety of this hydrogel and promote its clinical translation. In summary, this study demonstrates that the integrated design, relying on dynamic network architecture and multi-target synergistic mechanisms, provides novel design concepts and experimental evidence for developing smart wound dressings with dynamic-responsive properties, active pro-regenerative functions, and translational prospects.

## 4. Materials and Methods

### 4.1. Materials

Anhydrous calcium chloride, absolute ethanol, sodium chloride, stearyl methacrylate (C_18_), and polyvinyl alcohol 1750 ± 50 (PVA) were purchased from Sinopharm Chemical Reagent Co., Ltd. (Shanghai, China). Sodium hydroxide, sodium dodecyl sulfate (SDS), and manganese dioxide (MnO_2_) nanoparticles were obtained from Shanghai Macklin Biochemical Co., Ltd. (Shanghai, China). Streptozocin (STZ), sodium citrate, anhydrous citric acid, and paraformaldehyde powder were bought from Shanghai Aladdin Biochemical Technology Co., Ltd. (Shanghai, China). Penicillin-streptomycin was purchased from Beyotime Biotechnology Co., Ltd. (Shanghai, China). Triton X-100, 4’,6-diamidino-2-phenylindole (DAPI), sodium pentobarbital, bovine serum albumin (BSA), Hematoxylin and Eosin (H&E) Staining Kit, and Masson’s Trichrome Staining Kit were provided by Beijing Solarbio Science & Technology Co., Ltd. (Beijing, China). Dulbecco’s Modified Eagle Medium (DMEM) was purchased from Corning, and fetal bovine serum (FBS) was obtained from Biological Industries. 2-(2-Methoxy-4-nitrophenyl)-3-(4-nitrophenyl)-5-(2,4-disulfophenyl)-2H-tetrazolium monosodium salt (CCK-8) was bought from APExBIO. 3M transparent dressings were provided by 3M (Shanghai) International Trading Co., Ltd. (Shanghai, China). Rabbit monoclonal CD206 antibody, rabbit polyclonal CD31 antibody, and rabbit polyclonal CD86 antibody were purchased from Bioss. Goat anti-rabbit IgG H&L antibody was obtained from Abcam, and anti-fade fluorescence mounting medium was provided by Wuhan Boster Biological Technology Co., Ltd. (Wuhan, China).

### 4.2. Preparation of Composite Hydrogell

#### 4.2.1. Preparation of Methacrylated Hyaluronic Acid (HAMA)

Dissolve 5 g of sodium hyaluronate (HA) in 500 mL of deionized water. Under an ice-bath condition at 4 °C, add 12.5 mL of methacrylic anhydride (MAH) dropwise into the HA solution at a rate of 0.5 mL/min under continuous stirring. Upon completion of dropping, slowly add 10 mol/L sodium hydroxide (NaOH) solution into the mixture to adjust the pH to approximately 8.0, and allow the reaction to proceed at 4 °C for 12 h. Subsequently, add another 5 mL of MAH dropwise at 0.5 mL/min, adjust the pH to around 8.0, and carry out the reaction for another 12 h. Thereafter, dialyze the product at 40 °C for 5–7 days, centrifuge at 8000 rpm for 10 min to remove precipitates, collect the supernatant, freeze-dry for 2 days, and store at 4 °C. The dialysis membrane has a molecular weight cutoff (MWCO) of 3500 Da.

#### 4.2.2. Preparation of SDS-C_18_ Micelle Solution

An appropriate amount of sodium chloride was dissolved in deionized water to prepare a 0.8 mol/L NaCl solution, which was then heated to 50 °C. Sodium dodecyl sulfate (SDS) was added and stirred evenly, followed by the addition of C_18_. The mixture was continuously stirred for 1 h to obtain the micelle solution.

#### 4.2.3. Preparation of MC Multifunctional Composite Hydrogel

A total of 4 wt% HAMA solution, 15 wt% PVA solution, and 0.5 wt% MnO_2_ dispersion were prepared separately. In total, 4.0 g of 4 wt% HAMA solution was mixed with photoinitiator I2959, and the mixture was stirred in the dark until complete dissolution to obtain a HAMA/I2959 solution. The PVA solution, micelle solution, and HAMA/I2959 solution were thoroughly mixed at a preset mass ratio to obtain a HPS precursor solution. A total of 4 mL of HPS precursor solution was added to the MnO_2_ dispersion, vortexed, injected into a mold, and cross-linked under UV irradiation. The obtained hydrogel was subjected to multiple freeze–thaw cycles, then immersed in 1 mol/L CaCl_2_ solution for 2 h, and rinsed repeatedly with deionized water to fabricate the MC composite hydrogel. The entire operation was performed in the dark.

### 4.3. Performance Characterization of MC Composite Hydrogel

#### 4.3.1. Microstructure Characterization

Macroscopic hydrogel samples with dimensions of 2 cm (length) × 1 cm (width) × 0.4 cm (height) were fabricated. The microstructure of the samples was observed using scanning electron microscopy (SEM). The hydrogel was fully swollen, freeze-dried, and fractured with liquid nitrogen to expose a fresh cross-section, followed by gold sputtering for internal morphology observation. The freeze-dried micelle solution sample and MnO_2_ powder were treated with gold sputtering, and their particle morphology and dispersion state were observed.

#### 4.3.2. Injectability Evaluation

The hydrogel precursor solution was loaded into a syringe and extruded into specific patterns on the surface of a Petri dish to observe its moldability and shape retention ability. The precursor was also extruded into deionized water to evaluate its in situ cross-linking performance and structural stability in an aqueous environment.

#### 4.3.3. Electrical Conductivity and Self-Healing Property Evaluation

Disk-shaped hydrogel samples with a diameter of 20 mm and a thickness of 2 mm were prepared. The electrical conductivity and self-healing properties of the hydrogel were evaluated using an LED conductive circuit method. The two ends of a light-emitting diode (LED) were connected to the two ends of the hydrogel sample, respectively, to observe the electrical conductivity. The hydrogel was cut, and the fractured sections were closely fitted for healing at room temperature for a specified period. The healed hydrogel was reconnected to the LED circuit to observe the recovery of the conductive pathway.

#### 4.3.4. Adhesive Property Evaluation

Disk-shaped composite hydrogel samples with a diameter of 1 cm and a thickness of 0.2 cm were prepared. The adhesion characteristics of the hydrogel to various substrates were evaluated using a macroscopic lap method. The hydrogel was attached to the dorsal aspect of a volunteer’s knuckle to observe its dynamic compliance at different joint angles. The hydrogel was pre-adhered to the surface of a glass slide, flipped over to contact various abiotic materials including glass, metal, silicone, and leaves, and suspended vertically to observe its adhesion ability. Fresh isolated mouse tissues were collected, and the hydrogel was adhered to the tissue surface to evaluate the adhesion stability in air and underwater, respectively.

#### 4.3.5. Swelling Property Evaluation

Disk-shaped hydrogel samples with a diameter of 10 mm and a thickness of 2 mm were prepared. The swelling behavior of the hydrogel was investigated using the gravimetric method. Freeze-dried hydrogel samples were weighed and immersed in phosphate-buffered saline (PBS). The samples were taken out at preset time intervals, blotted dry to remove surface moisture, weighed, and photographed to record macroscopic morphological changes until swelling equilibrium was reached. The swelling ratio was calculated, and the swelling kinetic curve was plotted.

#### 4.3.6. Water Retention Property Evaluation

Disk-shaped hydrogel samples with a diameter of 10 mm and a thickness of 2 mm were prepared. The water retention capacity of the hydrogel was investigated using the gravimetric method. Hydrogel samples at swelling equilibrium were weighed and placed at room temperature. The samples were weighed at preset time intervals, the water retention rate was calculated, and the water retention kinetic curve was plotted.

#### 4.3.7. Mechanical Property Evaluation

Cylindrical hydrogel samples with a diameter of 10 mm and a thickness of 6 mm were uniformly prepared. A universal material mechanical testing machine was used to perform compression tests on cylindrical hydrogel samples. The stress–strain curves were recorded, and the fracture stress, fracture strain, and elastic recovery ability were analyzed. The results were expressed as mean ± standard deviation.

### 4.4. Biocompatibility Evaluation of MC Composite Hydrogel

In accordance with the National Standard ISO 10993 [52] series, the effects of the material on cell proliferation, blood components, and the physiological structure of core organs were comprehensively evaluated through in vitro cytotoxicity tests, in vitro hemolysis tests, and in vivo histocompatibility evaluation of major organs.

#### 4.4.1. Cytocompatibility Evaluation

Hydrogel extracts were prepared in accordance with the ISO 10993-12 [52] standard. Each group of hydrogels reaching swelling equilibrium was immersed in complete medium (DMEM) at a mass-to-volume ratio of 1 g: 10 mL and subjected to constant-temperature extraction in a water bath at 37 °C for 24 h. The culture medium was filtered through a 0.22 μm filter membrane to remove bacteria and monomer impurities and supplemented with 10% fetal bovine serum and 1% penicillin-streptomycin solution for subsequent use. The CCK-8 assay was used to detect the effects of the extracts on the viability of RAW 264.7, NIH/3T3, and HUVECs. The absorbance was measured after 1, 3, and 5 days of culture, and the cell viability was calculated.

#### 4.4.2. Hemocompatibility Evaluation

ICR mice were used as experimental subjects. Fresh anticoagulated whole blood was collected to isolate red blood cells (RBCs), which were washed and diluted to obtain an RBC suspension. The suspension was co-incubated with the hydrogel extracts, with normal saline and Triton X-100 set as the negative and positive controls, respectively. After centrifugation, the absorbance of the supernatant was measured, and the hemolysis rate was calculated to evaluate the effect of the material on blood components and determine its hemocompatibility.

#### 4.4.3. Histocompatibility Evaluation

SPF-grade ICR mice were housed in a standardized barrier environment. The mice were sacrificed after the wound intervention experiment, and the major organ tissues, including the heart, spleen, and lung, were collected. The tissues were fixed with paraformaldehyde, subjected to gradient dehydration and paraffin embedding, and sectioned for H&E staining. The structural integrity of the organs, myocardial and alveolar morphology, and inflammatory cell infiltration were observed under a light microscope to comprehensively evaluate the long-term in vivo histocompatibility of the hydrogel.

### 4.5. Evaluation of Antioxidant, Cell Migration-Promoting and Antibacterial Properties of MC Composite Hydrogel

#### 4.5.1. Antioxidant Property Evaluation

An extraction strictly at a solid–liquid ratio of 1 g: 10 mL for 24 h was performed, and the purified original extract was collected for subsequent use. The in vitro antioxidant activity of each group of hydrogels was evaluated using the DPPH free radical scavenging assay. Hydrogel samples were immersed in PBS for constant-temperature extraction for 24 h to obtain extracts, which were then mixed with DPPH ethanol solution and incubated in the dark. A blank control group and a sample control group were set up simultaneously and incubated with DPPH ethanol solution in the dark. The absorbance was measured, and the DPPH radical scavenging rate was calculated.

#### 4.5.2. Cell Migration-Promoting Effect Evaluation

The scratch wound assay was used to evaluate the effect of hydrogel extracts on the migration ability of NIH/3T3 fibroblasts and HUVECs. NIH/3T3 fibroblasts and HUVECs were cultured to a confluent state, and a scratch was made. The culture medium was replaced with hydrogel extracts for further incubation. Images of the scratch area were captured at 0, 12, and 24 h. ImageJ 1.54f software was used to calculate the change in scratch area, and the cell migration rate was obtained according to the formula to evaluate the wound repair potential of the material.

#### 4.5.3. Antibacterial Property Evaluation

Using *Staphylococcus aureus* and *Escherichia coli* as model strains, the antibacterial property and anti-biofilm activity of the hydrogel were comprehensively evaluated by the colony counting method, crystal violet staining method, and inhibition zone assay.

##### Colony Counting Method

Sterilized MC hydrogel samples were co-incubated with a standardized bacterial suspension at a concentration of 1 × 10^6^ CFU/mL. After incubation, the bacterial solution was subjected to a 10-fold serial gradient dilution. The dilutions at an appropriate gradient were spread on LB agar plates, and the colony-forming units (CFU) were counted after constant-temperature incubation to evaluate the bactericidal and bacteriostatic effects of the hydrogel on planktonic bacteria.

##### Crystal Violet Staining Method

Sterilized hydrogel samples and bacterial suspension were added to 12-well plates and subjected to stationary incubation at constant temperature for biofilm formation. After incubation, the residual bacterial solution was discarded, and the plates were washed with PBS to remove planktonic bacteria. The biofilms were fixed with methanol, stained with 0.1% crystal violet, and rinsed. The stained biofilms were then dissolved with glacial acetic acid, and the dissolved solution was transferred to a 96-well plate. The absorbance at 595 nm was measured using a microplate reader for quantitative analysis of the anti-biofilm efficacy of the hydrogel.

##### Inhibition Zone Assay

LB agar medium was prepared and autoclaved, and poured into plates when cooled to room temperature. 0.1 mL of bacterial suspension was uniformly spread on the agar surface, and the prepared disk-shaped hydrogel samples were gently placed in the center of the plates for constant-temperature incubation. After incubation, the formation of inhibition zones was observed and photographed to intuitively evaluate the antibacterial range and intensity of the hydrogel.

### 4.6. Animal Experiment in Diabetic Mice

#### 4.6.1. Establishment of Diabetic Mouse Model

The animal study protocol was approved by the Institutional Review Board (or Ethics Committee) of the Animal Center of the Medical Faculty, Ningbo University (protocol code 13411, date of approval 30 April 2024 ).

Male ICR mice aged 4–6 weeks with body weights of 20–40 g were selected to establish the diabetic mouse model. After one week of acclimatization, the mice received intraperitoneal injections of streptozotocin (STZ) working solution once daily for 5 consecutive days. During this period, drinking water was replaced with a 5% glucose solution. The mice were continuously fed for 1 week after the injection, and a random blood glucose level ≥ 16.7 mmol/L was set as the criterion for successful model establishment.

#### 4.6.2. Study on Skin Ulcer Repair in Diabetic Mice

The successfully modeled diabetic mice were randomly divided into six groups: blank control group, gauze group, PS group, HPS group, M group, and MC group. Mice in each group were anesthetized, and a full-thickness skin defect wound with a diameter of 6 mm was created on the dorsal region. The wounds were treated with the corresponding dressings, covered with a transparent film, and the dressings were changed every 2 days. The wound changes were observed and photographed at 0, 4, 8, 12, and 16 days after modeling, and ImageJ software was used to calculate the wound area and healing rate. At the end of the experiment (day 16), full-thickness skin tissue of the wounds was collected, fixed, embedded, and sectioned for H&E staining, Masson’s trichrome staining, and immunofluorescence histochemical staining, respectively. Tissue repair, collagen deposition, angiogenesis, and macrophage polarization were observed.

### 4.7. Statistical Analysis

GraphPad Prism 9.5.0 software was used for statistical analysis and graph plotting. All quantitative results are presented as mean ± standard deviation (Mean ± SD). At least three independent replicate samples were set for each experimental group to ensure data reliability. One-way analysis of variance (one-way ANOVA) followed by Tukey’s post hoc test was used for comparison among multiple groups, and an independent-samples t-test was used for comparison between two groups. A *p*-value < 0.05 was considered statistically significant.

## Data Availability

The data reported in this study are available from the corresponding author upon reasonable request.
